# Cohesin drives chromatin scanning during the RAD51-mediated homology search

**DOI:** 10.1126/science.adw1928

**Published:** 2025-12-04

**Authors:** Alberto Marin-Gonzalez, Adam T. Rybczynski, Namrata M. Nilavar, Daniel Nguyen, Andrew G. Li, Violetta Karwacki-Neisius, Roger S. Zou, Franklin J. Avilés-Vázquez, Masato T. Kanemaki, Ralph Scully, Taekjip Ha

**Affiliations:** 1. Howard Hughes Medical Institute and Program in Cellular and Molecular Medicine, Boston Children’s Hospital, Boston, MA, USA; 2. Department of Pediatrics, Harvard Medical School, Boston, MA, USA; 3. Department of Biology, Johns Hopkins University, Baltimore, MD, USA; 4. Department of Medicine and Cancer Research Institute, Beth Israel Deaconess Medical Center and Harvard Medical School, Boston, MA, USA; 5. Department of Medicine, Massachusetts General Hospital, Boston, MA, USA; 6. Department of Biophysics and Biophysical Chemistry, Johns Hopkins University, Baltimore, MD, USA; 7. Department of Chromosome Science, National Institute of Genetics, Mishima, Japan; 8. Graduate Institute for Advanced Studies, SOKENDAI, Mishima, Japan; 9. Department of Biological Science, Graduate School of Science, The University of Tokyo, Tokyo, Japan

## Abstract

Cohesin folds genomes into chromatin loops, whose roles are under debate. We find that double strand breaks (DSBs) induce *de novo* formation of chromatin loops, with the loop base positioned at the DSB site. These loops form only in S/G2 phases and occur during repair via homologous recombination (HR), concomitant with DNA end resection and RAD51 nucleoprotein filament assembly. RAD51 showed two-tiered accumulation around DSBs, with a broad (~Mb) domain likely arising from the homology search. This domain is regulated by cohesin unloader, is constrained by topologically associating domain (TAD) boundaries, and overlaps with chromatin regions reeled through the break-anchored loop, suggesting that loop extrusion regulates the homology search. Indeed, depletion of NIPBL results in reduced HR, and this effect is more pronounced when the HR donor is hundreds of kb from the DSB. Our data indicates that loop-extruding cohesin promotes the mammalian homology search by facilitating break-chromatin interactions within the damaged TAD.

## One-Sentence Summary

High spatiotemporal resolution analysis of double strand beak repair in 3D genome revealed the role of cohesin-driven loop extrusion in the homology search.

## INTRODUCTION

At the scale of hundreds of kilobases to a few megabases, mammalian genomes are organized into topologically associating domains (TADs) and chromatin loops (1, 2). These structures are dependent on the activity of the cohesin complex and are likely formed via a loop extrusion mechanism, whereby cohesin binds to chromatin and extrudes a loop until a convergent CTCF is found (3–7). Genomic loci that fall within the same TAD engage in more frequent mutual chromatin contacts than do loci from different TADs. Consequently, TADs have been proposed to play a role in modulating transcription by promoting intra-TAD and preventing inter-TAD enhancer-promoter interactions, with potential implications in health and disease (8–13).

The functions of TADs and chromatin loops have also been investigated in the context of DNA replication (14, 15), V(D)J recombination (16, 17) and DNA repair, particularly, the repair of DNA double-strand breaks (DSBs) (18–21). With respect to DSB repair, TAD boundaries appear to compartmentalize the chromatin response during the repair process, characterized by the formation of mega-basepair (Mb) scale chromatin domains composed of nucleosomes containing phosphorylated H2A.X (γH2AX) and γH2AX-associated repair proteins including MDC1, 53BP1 and BRCA1 (20, 21). ChIP-Seq analysis of the response to a site-specific mammalian DSB indicated that the γH2AX/MDC1/53BP1 domain is limited by TAD boundaries (20–22). An explanation for this compartmentalization was suggested by Arnould *et al*. (21). Based on the finding that DSBs induced by the AsiSI restriction enzyme form chromatin loop anchors, they proposed that H2AX is phosphorylated by the ATM kinase located at the DSB, and that the propagation of the γH2AX domain is mediated by cohesin-dependent extrusion of a break-anchored loop (21). Interestingly, recent work using live-cell imaging revealed that cohesin recruitment to DSB sites peaks significantly later than the recruitment of MDC1, the direct binding partner of γH2AX (23). Thus, break-anchored loop extrusion might have additional DSB response functions at stages later than the formation of γH2AX-dependent repair foci. Interestingly, in yeast, site-specific DSBs have also been shown to accumulate cohesin (24, 25) and have been observed to act as loop anchors (26). However, in this case the loops were found to form during DSB repair by homologous recombination (HR) and in a DNA end-resection-dependent manner (26). HR is a multistep pathway that entails: first, resection of each end of the DSB to generate long 3’ single-stranded (ss)DNA tails; second, loading of the RAD51 recombinase onto ssDNA to form the RAD51 nucleoprotein filament; third, the homology search—the process by which the RAD51 nucleoprotein filament searches for a homologous donor, culminating in RAD51-mediated strand exchange; fourth, repair synthesis and termination of the HR reaction.

In summary, although several functions have been proposed for break-anchored chromatin loops in DSB repair, their role remains poorly understood. Here, we investigated this question using a combination of time-course ChIP-Seq, time-course Hi-C and quantitative assays of HR.

## RESULTS

### Cas9 breaks are anchors for cohesin loop extrusion.

**Figure 1.**
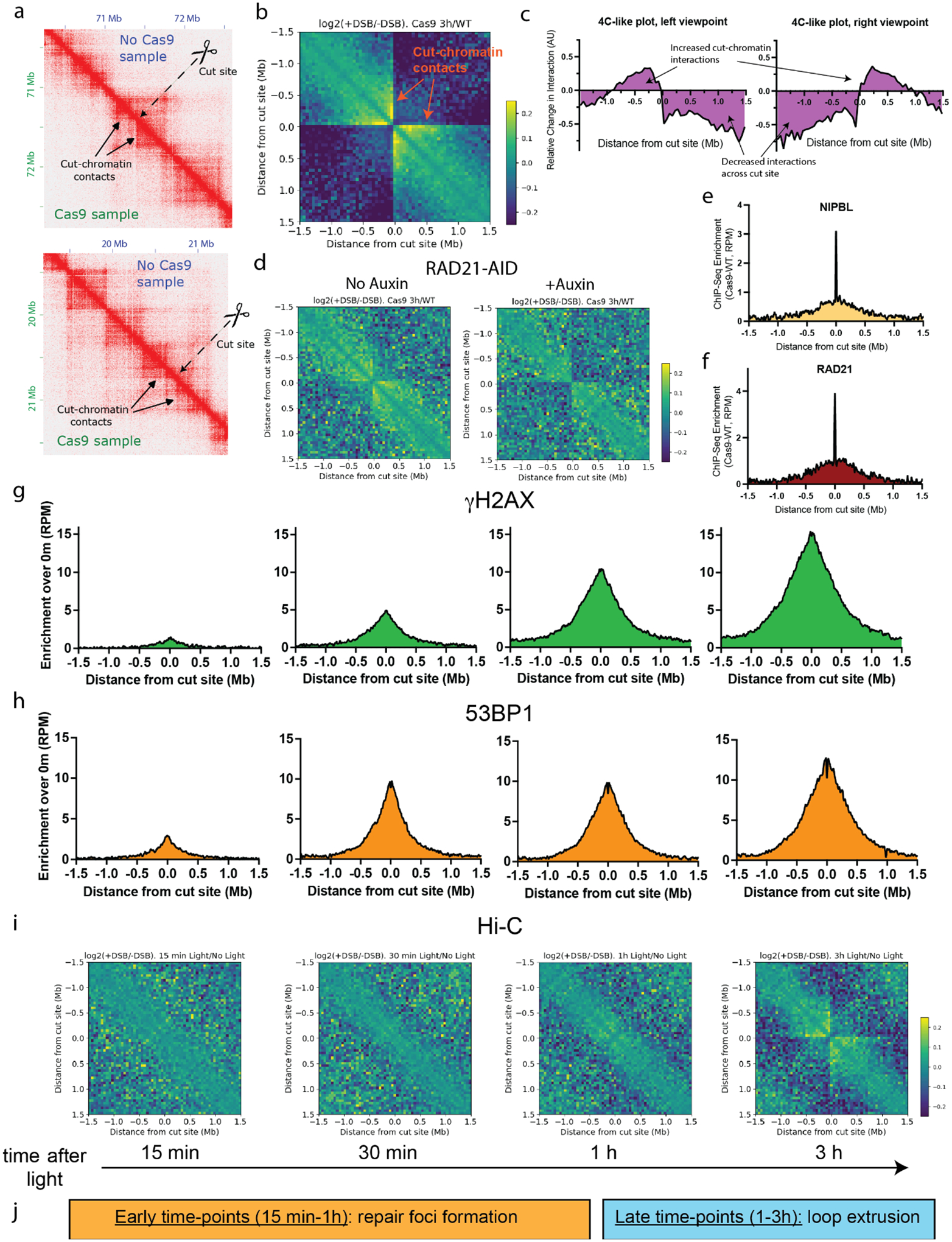
Break-anchored chromatin loops at Cas9 breaks. **a**, High-resolution (5 kb) Hi-C maps around two representative AluGG cut sites (in chr9, top; and chr14, bottom) in HEK293T. Untreated samples are shown in the top right triangle, Cas9-treated samples are shown in bottom left. **b**, Average log2 ratio of chromatin contacts around the 100 AluGG cut sites with the strongest MRE11 enrichment. Hi-C matrices were retrieved at 50 kb resolution in a window of 3 Mb, centered at each MRE11 peak. **c**, 4C-like plots were computed from high-resolution Hi-C matrices around the top 100 MRE11 peaks using a left and right viewpoint with respect to the cut site for the Cas9-treated and undamaged sample. **d**, Cas9-induced chromatin contacts in HCT116-RAD21-AID2 cells with no drug (left) or with auxin treatment (right). Differential Hi-C contacts were computed and plotted as in panel b. **e, f**, Cas9-induced NIPBL and RAD21 ChIP-Seq enrichment. ChIP-Seq profiles for NIPBL and RAD21 were averaged around the 126 AluGG on-target sites in Cas9-treated and untreated cells. The resulting untreated profile was subtracted from the Cas9-treated profile. RPM: reads per million. Bin size: 5 kb. **g-i**, Time-course γH2AX (g), 53BP1 (h) ChIP-Seq and Hi-C (i). HEK293T cells were treated with Cas9 RNP with caged AluGG gRNA for 12h, followed by light-induced Cas9 activation and harvest at 0 min, 15 min, 30 min, 1 h and 3 h. ChIP-Seq profiles were computed as described in panel **e, f**, and average Hi-C profile was obtained as in panel **b**, using the 0 min (no-light) control sample as reference. **j**, A proposed timeline.

To study DSB-induced changes in the 3D genome, we induced DSBs using our recently developed multi-target CRISPR system, where a degenerate guide RNA (gRNA), or multi-target gRNA (mgRNA), directs a Cas9 nuclease to induce DSBs at >100 endogenous on-target sites (27). We nucleofected HEK293T cells with ribonucleoprotein complexes (RNPs) composed of purified Cas9 preassembled with the AluGG mgRNA, a gRNA we previously characterized (27), and performed *in situ* Hi-C (2) 3 h after delivery of the CRISPR/Cas9 RNP as well as in control, untreated cells. For each condition, Hi-C was done in biological replicates and, after confirming strong reproducibility (Fig. S1), we merged replicates totaling ~1B contacts per condition, which allowed us to build high-resolution Hi-C contact maps. Visual comparison of Hi-C maps around select Cas9 breaks revealed ‘stripes’ that emanated from the predicted location of the Cas9 break (Fig. 1a). These stripes, which represent interactions between the break site and neighboring loci, were absent in the untreated control sample, suggesting that localized DSB-chromatin interactions arise *de novo* because of Cas9 activity.

To better visualize Cas9-induced changes in chromatin contacts, we computed the log2 of the ratio between the contacts in the Cas9 treated sample and the untreated sample averaged among the 100 best cleaved sites, as determined from previous MRE11 ChIP-Seq (27). The resulting differential Hi-C map revealed increased interactions between DSB sites and their neighboring chromatin, represented as cross-shaped stripes (Fig. 1b), consistent with previous studies (21, 28) and with our inspection of individual cut sites (Fig. 1a). We further characterized these DSB-chromatin interactions by computing differential 4C-like plots using the regions adjacent to the breaks as viewpoints (Fig. 1c). DSB-chromatin interactions spanned a distance of ~1 Mb in both directions from the break. Interactions between loci spanning the DSB site were diminished, suggesting that Cas9-induced DSBs acquire chromatin insulation properties, reminiscent of TAD boundaries.

Cohesin has been implicated in the formation of chromatin loops during repair of DSBs induced by the AsiSI restriction enzyme (21). To test whether cohesin is involved also at Cas9-induced breaks, we delivered multi-target CRISPR RNP into an HCT116 cell line with RAD21 tagged for auxin induced degradation (AID) (29) and performed Hi-C 3h later. To degrade RAD21, we added auxin 1h prior to RNP nucleofection. RAD21 degradation abolished the DSB-chromatin contacts, demonstrating that cohesin is instrumental in the formation of these structures (Fig. 1d and Fig. S2). Similar results were obtained when depleting RAD21 via siRNA in HEK293T cells (Fig. S2). Finally, the loop extruding cohesin subunit NIPBL was recruited to Cas9 breaks in HEK293T cells, as determined by ChIP-Seq, as were two additional cohesin subunits, RAD21 and the DNA-damage phosphorylated form of SMC1 (pSMC1) (Fig. 1e, 1f and Fig. S3), further supporting a direct involvement of cohesin. We conclude that Cas9-induced DSBs interact with their neighboring loci by a cohesin-driven mechanism, possibly involving loop extrusion.

### Break-anchored loops form after the formation of the γH2AX domain.

Previous work reported the existence of break-anchored chromatin loops at restriction enzyme-induced DSBs, and the authors proposed that a cohesin-based loop extrusion mechanism is required for formation of the ~1 Mb DNA repair chromatin domain marked by γH2AX (21). To investigate the relative timing of the break-anchored chromatin loops and γH2AX domain formation, we performed a time-course experiment using a light-activated, very fast (vf) CRISPR version of our multi-target system (27, 30). In parallel to time-resolved ChIP-Seq for γH2AX and 53BP1 – two major factors known to decorate chromatin within the TAD that contains the DSB (31), we performed time-resolved Hi-C, capturing events at 15 min, 30 min, 1 h and 3 h after CRISPR activation (Fig. 1g–i). γH2AX and 53BP1 were detected as early as 15 min (Fig. 1g, h and Fig. S4), consistent with rapid establishment of these responses (30, 31). The γH2AX signal spread over the entire time-course studied, with the largest changes observed in early (15 min to 1 h) time points, whereas 53BP1 roughly saturated at 30 min. In contrast, Hi-C revealed no break-anchored chromatin loops at time points earlier than 1 h, and these loops showed a robust signal only at 3 h (Fig. 1i and Fig. S4). These findings are consistent with recent live-cell imaging measurements showing that cohesin recruitment to a DSB peaks after the formation of MDC1 foci (23).

In summary, we find that break-anchored chromatin loops reach peak intensity well after the γH2AX chromatin domain has formed (Fig. 1j), suggesting that the break-anchored loop primarily operates in later stages of the DSB response.

### Break-anchored loops form during DSB processing for homologous recombination.

**Figure 2.**
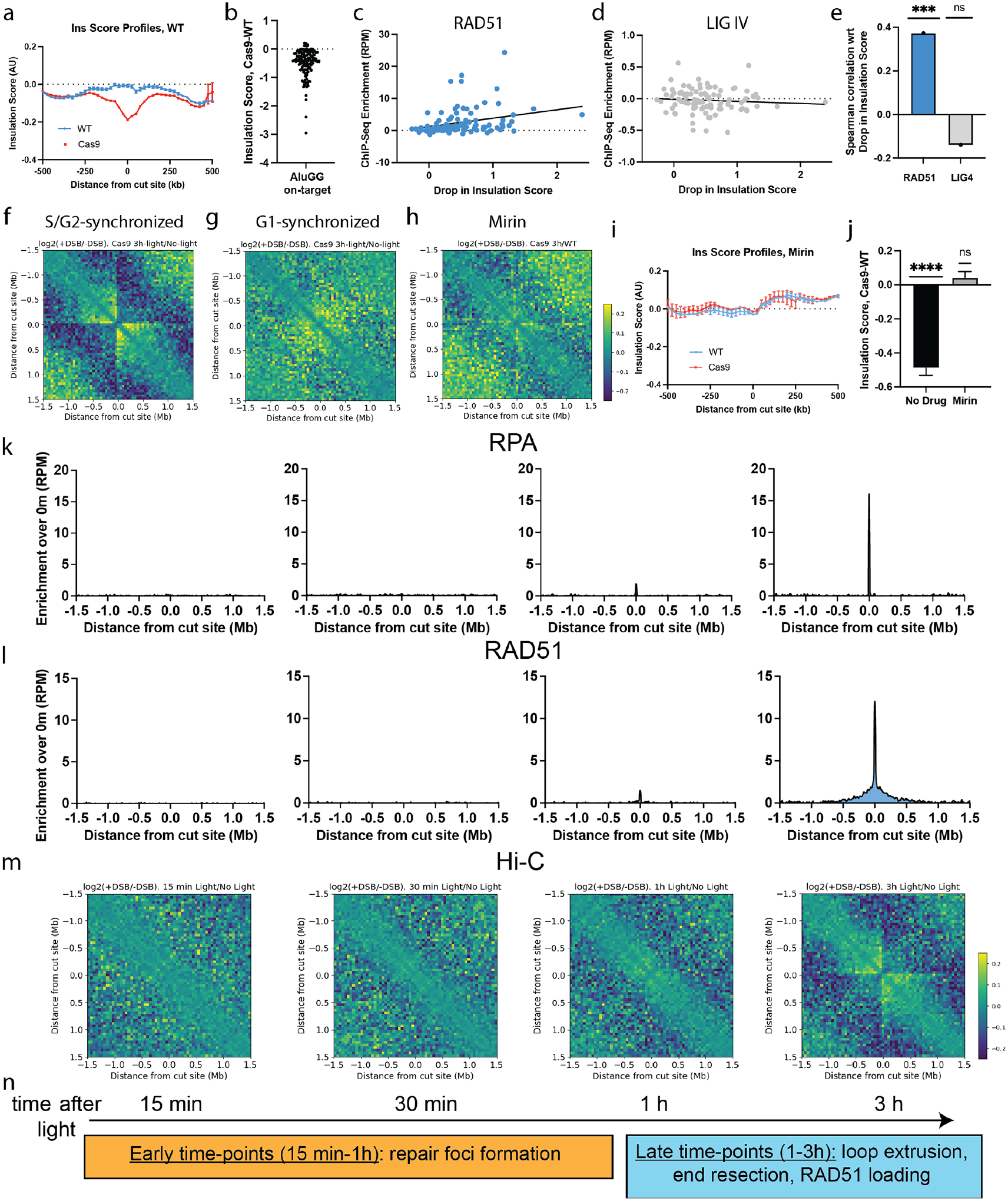
Break-anchored chromatin loops are formed during homologous recombination. **a**, Insulation score analysis around Cas9 breaks. Whole-chromosome Hi-C matrices were retrieved at 25 kb resolution and insulation score was then computed for each chromosome using the matrix2insulation script from the cworld package (32). Profiles were extracted in a window of 1 Mb around each of the 126 AluGG on-target sites and averaged. **b**, Change in insulation score upon Cas9 treatment was computed in a window of 50 kb centered around each AluGG on-target site. Note: reduced insulation score implies increased insulation. **c, d**, ChIP-Seq enrichment of RAD51 (c) and DNA ligase IV (d) vs drop in insulation score upon Cas9 treatment per cut site. Black lines are linear fits. **e**, Spearman correlation coefficient from (c) and (d). **f, g**, Averaged differential Hi-C contact maps in HEK293T cells synchronized in S/G2 (f) or G1 (g). **h, i**, Averaged differential Hi-C contact maps around best cut sites (h) and insulation score profiles around on-target AluGG sites (i) in cells treated with Mirin. **j**, Cas9-induced change in insulation score around AluGG on-target sites in cells without drug or treated with Mirin. Error bars are the standard error of the mean. Statistical significance with respect to null hypothesis was obtained using one sample *t*-test. **k-m**, Time-course of RPA (k), RAD51 (l) ChIP-Seq and Hi-C (m). Hi-C plots are reproduced from Fig. 1i for each comparison. RPA and RAD51 ChIP-Seq profiles were computed as in Fig. 1g, h. **n**, A proposed timeline.

To examine the role of break-anchored loops at late stages of DSB repair we further analyzed our Hi-C data on a site-by-site basis to determine whether Cas9 sites with stronger changes in chromatin contacts are prone to undergo a particular repair pathway. In interphase nuclei, chromatin loops compartmentalize the genome into TADs; by convention, a *lower* ‘insulation score’ metric at a TAD boundary indicates a *higher* level of insulation across the boundary (1, 32). Using our high-resolution Hi-C data, we computed average insulation score profiles around the 126 AluGG gRNA on-target sites and found that Cas9 treatment reduces the insulation score (i.e., increases the degree of insulation across the DSB) in comparison to untreated controls (Fig. 2a). A site-by-site analysis revealed a decrease in insulation score in 117 out of 126 on-target sites upon DNA damage, indicating that the observed change in average insulation score profiles does not stem from a few breaks, but rather, is a general DNA repair phenomenon (Fig. 2b). This drop in insulation score could result from two mutually nonexclusive phenomena: (1) Cas9 breaks disrupt the chromatin polymer and might bring about physical separation of the DNA ends; (2) Cas9 breaks might act as *de novo* TAD-like boundaries.

To determine which repair pathway is dominant at each cut site, we performed ChIP-Seq for DNA ligase IV (LIG4) and RAD51, which have been used to classify DSBs as non-homologous end-joining (NHEJ) or HR prone, respectively (33). Consistent with both pathways being operative at Cas9 breaks, the average ChIP-Seq profiles revealed a damage-induced enrichment of both LIG4 and RAD51 in the vicinity of Cas9 on-target sites (Fig. S5). We thus computed the Cas9-induced ChIP-Seq enrichment on a site-by-site basis and computed the Spearman correlation *r* between this ChIP-Seq enrichment and the drop in insulation score around the 100 best cleaved Cas9 DSB sites. RAD51 showed a significant positive correlation (*r*=0.37) with the damage-induced decrease in insulation score, whereas LIG4 showed a weak, non-significant, negative correlation (*r*=−0.17) (Fig. 2 c–e). Thus, DNA breaks that are prone to undergo HR are associated with stronger insulation properties. Notably, the ChIP-Seq signal intensity for each of three cohesin subunits—RAD21, pSMC1 and NIPBL—showed a significant positive correlation with the DSB-induced drop in insulation score (Fig. S6).

Based on these findings, we hypothesized that break-anchored loops require processing of the DSB for repair by HR. To test this hypothesis, we implemented a protocol to induce Cas9 breaks at defined cell cycle stages, by exploiting the vfCRISPR system (30). In short, cells were synchronized using thymidine blocks and were then electroporated with a Cas9-caged gRNA RNP at the time of release from the block (Fig. S7). Cells were then exposed to 365 nm light to activate the caged vfCRISPR at defined time points following release from the block, corresponding to well-defined synchronized cell cycle stages (Fig. S8, 9). Using this approach, we activated vfCRISPR in cells synchronized in G1 by shining light 15h after release from a double thymidine block (corresponding to ~91.7% of cells in G1 at the time of inducing breaks, Fig. S9), and in S/G2 phases by shining light 6h after release from a single thymidine block (~84% of cells in S/G2, Fig. S8). We then performed Hi-C 3h after DSB induction and compared the results with similarly synchronized, nucleofected cells that were not exposed to vfCRISPR-activating light. Induction of breaks in cells synchronized in S/G2, which are able to perform HR, recapitulated the break-centered Hi-C stripes observed in unsynchronized cells (Fig. 2f and Fig. S10). Strikingly, G1-synchronized showed no appreciable change in chromosome contacts in response to Cas9-induced DSBs (Fig. 2g and Fig. S10). We confirmed that DSB induction levels were comparable between G1- and late S/G2-synchronized cells (Fig. S11). Given that HR is active in late S/G2 and inactive in G1, our results suggest that break-anchored loop formation occurs in relation to DSB processing for HR.

To further test the relationship between break-anchored loop formation and HR, we performed multi-target CRISPR delivery and Hi-C in the presence of mirin, a drug that suppresses HR by preventing the MRE11 exonuclease-mediated step of DNA end resection (34). Notably, treatment with mirin greatly reduced the strength of the stripes (Fig. 2h and Fig. S12) and eliminated insulation score changes upon Cas9 breaks (Fig. 2 i, j), suggesting that break-anchored loops and their associated insulation properties are formed during HR in a DNA end resection-dependent manner.

### Time-course measurements support a role for break-anchored loop extrusion in HR.

We investigated the temporal relationships between DNA end resection, RAD51 loading and break-anchored loop extrusion by performing time resolved ChIP-seq of RPA and RAD51 ChIP-seq at vfCRISPR-induced DSBs.

RPA and RAD51 were undetectable within the first 30 min after DSB induction, revealing a strong, Cas9-induced enrichment only at 3 h (Fig. 2k, l and Fig. S4), with RPA ChIP-seq accumulating around a narrow window (~5 kb) centered at the cut site, and the RAD51 signal propagating to distances of several hundreds of kilobases away from the break. Thus, in our multi-target vfCRISPR system, resection and RAD51 loading occur at relatively late time points (> 1 h). Strikingly, the time course of RPA and RAD51 recruitment matched closely the dynamics of loop formation captured by time-resolved Hi-C (Fig. 2m and Fig. S4). This temporal coordination further supports a causal link between specific steps of HR and the formation of break-anchored loops.

In conclusion, our time-resolved experiments reveal two different time-scales during Cas9-induced DSB repair (Fig. 2n): first, γH2AX and 53BP1 chromatin responses first become detectable at early time points (15 min – 1 h), whereas DNA end resection, RAD51 loading, and break-anchored loop formation occur at later time points (1–3 h).

### A broad, RAD51 chromatin domain reflects the homology search.

**Figure 3.**
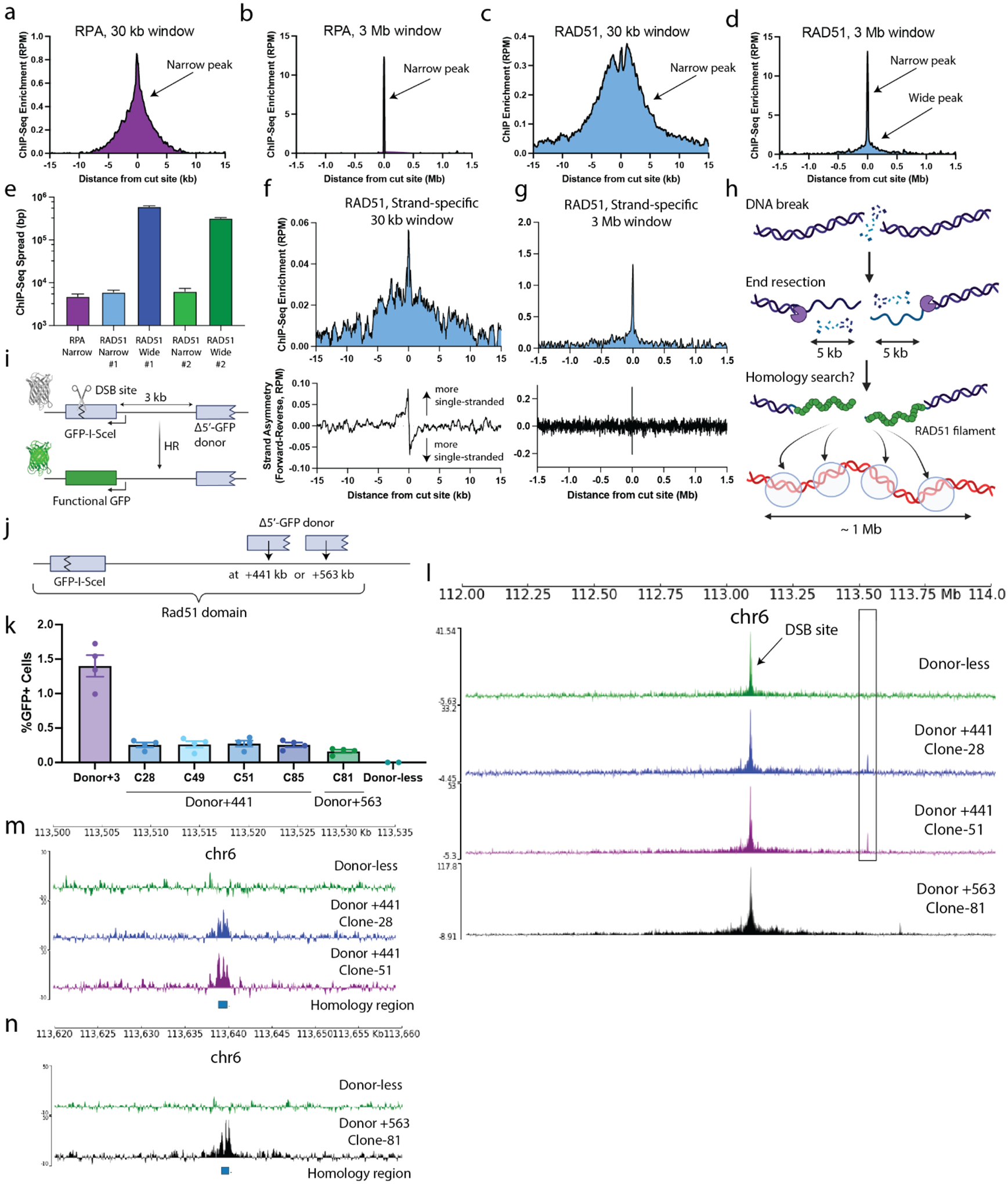
Broad RAD51 ChIP-Seq profiles inform on homology search. **a-d**, Average RPA (a, b) and RAD51 (c, d) ChIP-Seq profiles around AluGG on-target sites in a window of 30 kb (a, c) and 3 Mb (b, d). Arrows mark narrow (RPA, RAD51) and wide (RAD51) peaks. Bin size is 50 bp (a, c) and 5 kb (b, d). **c**, Average width of RPA and RAD51 peaks. Error = standard error of the mean. **f, g**, Strand specific ChIP-Seq of RAD51. Top panels are regular ChIP profiles (30 kb and 3 Mb windows). Bottom panels show the strand asymmetry, computed as the difference between the forward and the reverse reads. Bin size is 50 bp (panel f), 5 kb (panel g, top) and 1 kb (panel g, bottom). **h**, Cartoon depicting the interpretation of the broad RAD51 profile as a measure of homology search. **i**, Schematic of the HR-GFP reporter system to measure DSB-induced HR. *GFP-I-SceI* heteroallele harbors a site for DSB induction. HR repair via use of a 5’-Truncated (Δ5’-)*GFP* donor ~3 kb away from the DSB (here termed ‘Donor+3’) generates WT GFP that can be measured in flow cytometry. **j**, Schematic of the reporter system designed to study HR using distant donors within the TAD. A monoclonal mES cell clone was generated with a single *GFP-I-SceI* copy at *Rosa26* on Chr6. Then, derivative monoclonal lines were made with the *Δ5’-GFP* HR donor at +441 kb or +563 kb from the *GFP-I-SceI* copy (Figs. S17–S20). **k**, HR readout for Donor+3, four Donor+441 clones, a Donor+563 clone, and the donor-less clone. **l**, RAD51 ChIP-Seq profile in donor-less cells (top), in two of the Donor+441 clones and the Donor+563 clone. **m**, Zoom-in of (l) showing the RAD51 signal over *Δ5’-GFP* in the donor-less clone vs. two Donor+441 clones. The donor-less clone ChIP-Seq data in panels m, l was aligned to a genome containing a +441kb donor, but did not generate a signal over the donor locus. **n**, same as m but showing the Donor+563 clone and its associated *Δ5’-GFP* site.

To further explore HR events, we performed ChIP-seq for RPA and RAD51 3 h after delivery of regular, non-caged AluGG gRNA. The resulting RPA ChIP-Seq profile after delivery of regular AluGG gRNA showed a peak spanning a width of 4.8 ± 0.7 kb, centered at the cut site (Fig. 3a, b), a finding that is consistent with previous reports showing that DNA end resection extends for a few kilobases from the DSB (35). The RAD51 profile showed a peak of similar width of 6.1 ± 0.7 kb, which we attribute to ssDNA that is coated by the recombinase (Fig. 3c, d). In addition to this peak, the average RAD51 ChIP-Seq signal spread to a broad domain of 600 ± 30 kb in size (Fig. 3d, e), consistent with our time-course measurements (Fig. 2l). Visual inspection of RPA and RAD51 ChIP-Seq signals at individual AluGG cut sites corroborated the presence of these broad RAD51 domains that extend beyond the main, narrow peak (Fig. S13). An alternative RAD51 antibody that binds a different epitope gave a similar average ChIP-Seq profile (Fig. S14) with indistinguishable widths for both the narrow peak (P=0.85) and the broad RAD51 domain (P=0.54) (Fig. 3e).

RAD51 binds both ssDNA and dsDNA (36). We thus hypothesized that the broad RAD51 domain, which lacks RPA binding, represents RAD51-dsDNA interactions. To test this, we performed strand specific ChIP-seq (37) at 3 h. The RAD51 strand-specific ChIP-Seq profile showed similar qualitative features to the regular ChIP-Seq profile, including a peak at the cut site and a broad chromatin domain (Fig. 3f, g and Fig. S15). Analysis of strand asymmetry at each chromosomal location revealed that the extent of RAD51-ssDNA binding is constrained to a region of ~2 kb centered at the break, with the expected switch in polarity on either side of the DSB, reflecting RAD51 loading onto the resected 3’ ssDNA DNA ends (37). In contrast, at distances >2.5 kb from the DSB, strand asymmetry was lost, implying a RAD51-dsDNA binding mode.

Studies *in vitro* suggest that, during the homology search, the Rad51-ssDNA nucleoprotein filament interacts with a dsDNA template and scans it locally through a one-dimensional random walk, as part of the homology search (38). If the dsDNA is homologous to the RAD51 nucleoprotein filament, HR proceeds with formation of a D-loop. In contrast, the RAD51 nucleoprotein filament will dissociate from a non-homologous dsDNA (38). In yeast, Rad51 ChIP-Seq analysis at a site-specific DSB was suggested to reflect the extent of the homology search, with the Rad51 ChIP-Seq profile spanning a broad chromatin domain that represents Rad51-dsDNA interactions during chromatin scanning (39).

We asked whether – similar to the yeast case – the broad RAD51 domain that we observe in mammalian cells has features characteristic of homology search (Fig. 3h), in particular, whether it shows local accumulation at an appropriate HR donor (38, 39). We adapted a previously described HR reporter, which contains a *GFP* heteroallele (*GFP-I-SceI*) comprised of a full length *GFP* gene that is interrupted by a target site for the homing endonuclease I-SceI, and a 5’ truncated (*Δ5’*-) *GFP* recombination donor situated ~3 kb (here termed ‘Donor+3’) from the *I-SceI* site (Fig. 3i) (40). HR induced by a site-specific DSB localized to the *I-SceI* site is thus quantified by the production of GFP^+^ cells. Using a monoclonal mouse embryonic stem (mES) cell line that contains a single-copy HR reporter at the *Rosa26* locus of Chr6, we first removed the *Δ5’-GFP* donor *via* a CRISPR/Cas9-induced deletion. We nucleofected a Cas9-gRNA RNP targeted to the *I-SceI* site to induce a DSB within *GFP-I-SceI* in these “donor-less” cells, and ChIP-seq showed a broad RAD51 domain extending approximately −800 kb to +700 kb around the break site (Fig. S16). To determine whether the Rad51 signal is enriched over the HR donor during a successful homology search, we generated a new *Δ5’-GFP* donor containing select silent point mutations (conserving the amino acid sequence of GFP) sufficient to distinguish it from *GFP-I-SceI* during ChIP-seq sequence alignment. We targeted the new *Δ5’-GFP* donor to positions +441 kb (“Donor+441”) or +563 kb (“Donor+563”) telomeric to the *Rosa26*-located *GFP-I-SceI* (Fig. 3j), generating four independent Donor+441 clones and one Donor+563 clone in which a single, intact *Δ5’-GFP* donor was targeted to the same copy of Chr6 as the *Rosa26*-located *GFP-I-SceI* heteroallele (see Methods and Fig. S17–S20). *I-SceI*-induced HR in Donor+441 clones and the Donor+563 clone was readily detected but was less efficient than in the original Donor+3 HR reporter cell line (Fig. 3k and Fig. S20; see Methods). Consistently, the donor-less clone showed no detectable HR levels (Fig. 3k).

We then used Cas9-gRNA RNP nucleofection to induce a DSB at *GFP-I-SceI* in two Donor+441 clones and the Donor+563 clone and performed RAD51 ChIP-Seq 3 h after Cas9 delivery. Of note, in addition to the above-noted broad RAD51 distribution, we observed an additional, narrow and more intense peak at the location of the HR donor, at either +441 kb or +563 kb (Fig. 3l and Fig. S21). This enhanced RAD51 signal over the *Δ5’-GFP* HR donor likely represents the result of a successful homology search by the RAD51 nucleoprotein filament(s) derived from the DSB at *GFP-I-SceI*. A zoomed-in view revealed that this RAD51 peak overlies the *Δ5’-GFP* donor (Fig. 3m). We quantified the relative RAD51 signal at the donor relative to the DSB and found that it was higher in the +441 kb clones than the +563 kb (Fig. S22), consistent with the former showing higher HR levels (Fig. 3k).

To exclude the possibility that the RAD51 ChIP-Seq peak at the *Δ5’-GFP* donor might be a read alignment artifact due to the high sequence similarity between the *Δ5’-GFP* donor and *GFP-I-SceI*, we aligned the RAD51 ChIP-Seq data derived from the “donor-less” HR reporter clone to a reference genome that includes the *Δ5’-GFP* donor (at positions +441 or +563) and observed no specific Rad51 enrichment over the *Δ5’-GFP* donor (Fig. 3l, m and Fig. S21). Thus, RAD51 ChIP-seq reads that faithfully map to the *GFP-I-SceI* heteroallele at *Rosa26* are not prone to artifactual alignment with the *Δ5’-GFP* heteroallele. We confirmed this conclusion by manually reviewing each of the 1223 RAD51 ChIP-Seq reads that mapped to the +441 donor *GFP* heteroallele. We then asked whether the RAD51 peak over the +441 donor is single-stranded or double-stranded. To this end, we performed strand-specific RAD51 ChIP-Seq on one of the +441 donor clones 3 hours after DSB induction. In contrast to the RAD51 signal at the DSB site, the RAD51 signal over the +441 kb donor was predominantly double-stranded, implying that the major mode of RAD51 DNA binding to the +441 donor is *via* duplex DNA (Fig. S23). Interestingly, however, there was a mild degree of strand asymmetry over the donor, but the polarity of the DNA ends was opposite to that seen at the DSB site (Figs. S23b and S23c). Such a pattern of reversed strand symmetry could reflect nascent DNA synthesis during gene conversion using the +441 donor as template, thereby extending the invading ssDNA strand beyond the original site of the DSB to align it with the half of the donor *GFP* sequence homologous to the other side of the DSB (Fig. S24). In conclusion, the broad RAD51 domain shows local enrichment over the *Δ5’-GFP* HR donor, reflecting a successful homology search and, possibly, the onset of nascent strand extension at the +441 donor site.

### Loop extruding cohesin promotes RAD51-chromatin interactions within the damaged TAD.

**Figure 4.**
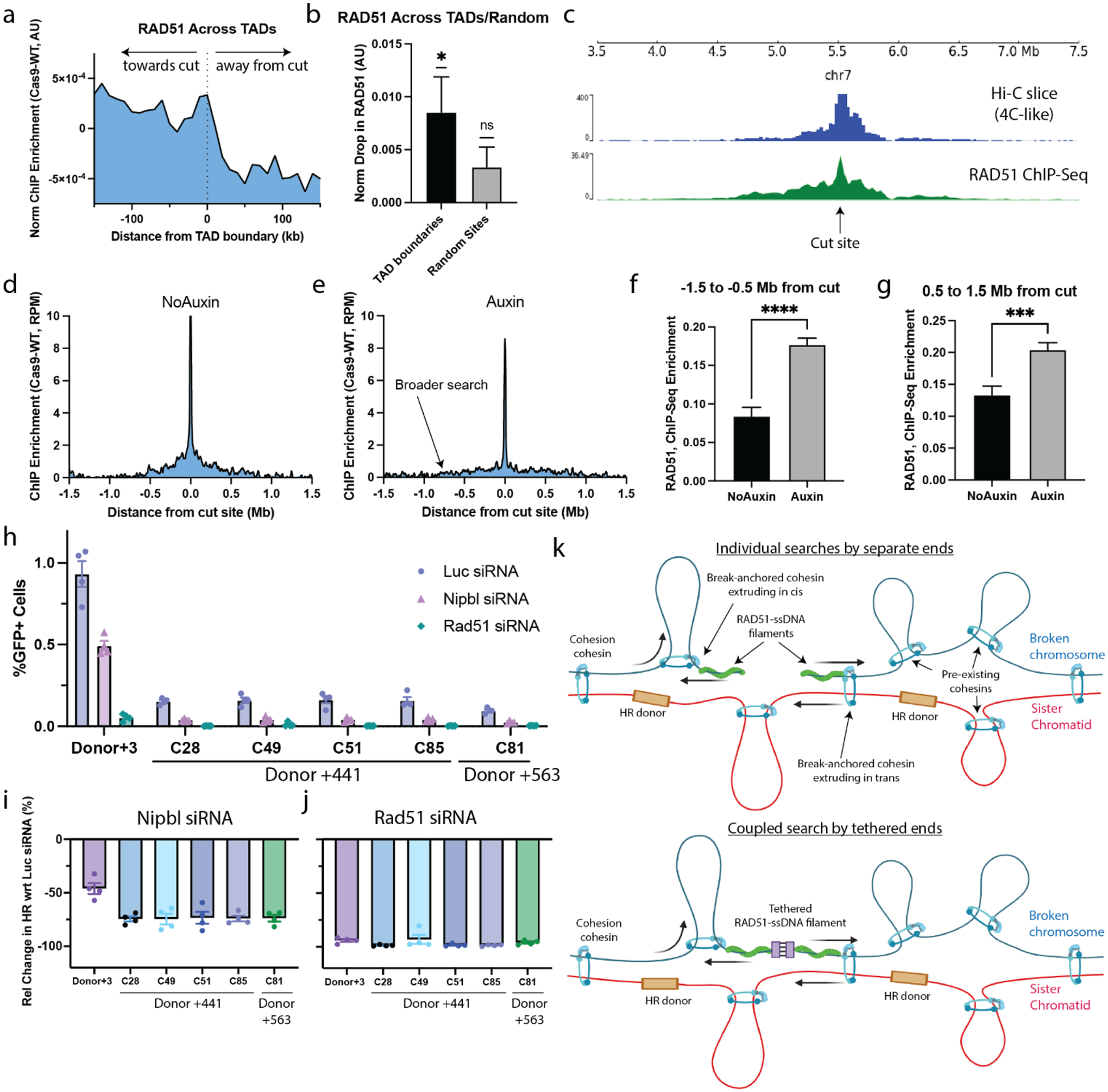
Loop-extruding cohesin regulates homology search and overall HR efficiency. **a**, RAD51 ChIP-Seq enrichment normalized and averaged around TAD boundaries that lie 200 kb to 700 kb away from the 100 best Cas9 cut sites. **b**, Normalized RAD51 drop at TAD boundaries and random sites (picked at distances between 200 kb and 700 kb away from cut sites). Error = standard error of the mean. One-sample *t*-test was done to quantify significance from null hypothesis. 155 TAD boundaries and 389 random sites were considered for the analysis. **c**, RAD51 ChIP-Seq profile and 4C-like profile at ACTB gene in HEK293T. 4C-like plot was obtained from the high-resolution Hi-C data in undamaged cells using a 100 bp region centered at the cut site as viewpoint. **d, e**, Average RAD51 ChIP-Seq profiles after Cas9-AluGG-gRNA-induced DSBs in HCT-WAPL-AID2 cells without (d) or with (e) auxin. **f, g**, Average change in RAD51 ChIP-Seq enrichment was obtained at regions −1.5 to 0.5 Mb (f) or 0.5 to 1.5 Mb (g) away from the cut. Error = the standard error of the mean. Unpaired *t*-test was done to quantify significance between the two conditions. **h**, HR-GFP assays were performed on the monoclonal cell lines from Fig. 3k after treatment with siRNA against control luciferase (Luc), Nipbl or Rad51. **i, j**, Quantification of h, showing changes in HR following depletion of Nipbl- (i) or Rad51-induced (j), normalized to Luciferase siRNA HR levels. **k**, Cartoon depicting different mechanisms for cohesin-driven chromatin scanning during homology search.

Our time-course data from Fig. 2 indicated that formation of the broad RAD51 domain occurs approximately contemporaneously with the break-anchored chromatin loops, raising the possibility that the break-anchored loops might participate in the homology search. Given that loops are often delimited by TADs (2), we evaluated the effect of TAD boundaries on the spread of the broad RAD51 ChIP-Seq signal obtained in HEK293T subjected to AluGG DSBs (data from Fig. 3c). We computed the averaged, RAD51 ChIP-Seq enrichment around TAD boundaries that lie between 200 kb and 700 kb away from the 100 most efficiently cleaved AluGG cut sites (according to MRE11 ChIP-Seq) and noted a sharp decrease in RAD51 signal at the location of the TAD boundary (Fig. 4a, b). As control, we performed the same analysis around random, non-TAD boundary sites and observed no significant reduction in RAD51 signal (Fig. 4b). Therefore, at least some TAD boundaries correlate with the extent of the broad RAD51 peak, possibly constraining the homology search to the damaged TAD. This finding is in agreement with recent work reporting broad, TAD-delimited RAD51 chromatin domains in single cells after induction of site-specific DSBs (22). To explore the link between chromatin structure and the broad RAD51 peak at the level of a single locus, we treated HEK293T cells with a Cas9 complexed with an ACTB-targeting gRNA, previously shown to trigger Cas9 cleavage with high efficiency (30), and performed RAD51 ChIP-Seq. Interestingly, the resulting ChIP-Seq profile showed a high similarity with the chromatin interaction profile in untreated cells (obtained from our high-resolution Hi-C data using the cut site as viewpoint) (Fig. 4c). Similar findings regarding the similarity in RAD51 signal and chromatin interactions were obtained using an additional gRNA targeting the MYC gene (Fig. S25). Thus, TAD boundaries, and possibly other aspects of chromatin architecture correlate with the extent of the broad RAD51 peak.

Next, we tested the role of chromatin loops on the extent of the broad RAD51 domain. WAPL unloads cohesin from chromatin and acute WAPL degradation results in elongated loops, likely by extending cohesin’s residence time on chromatin (41). To manipulate WAPL levels, we employed a previously characterized HCT116 cell line, hereafter HCT116-WAPL-AID2, where WAPL is endogenously tagged with a degron that permits fast, acute degradation upon the addition of auxin with minimal leakage (15, 42). We delivered multi-target CRISPR into HCT116-WAPL-AID2 cells and compared the extent of spread of the broad RAD51 peak in the presence and absence of auxin. Consistent with a role of loop extrusion in the homology search, WAPL loss resulted in a broader RAD51 ChIP-Seq domain (Fig. 4d, e and Fig. S26). Specifically, subtraction of the RAD51 profile in the absence (Auxin) and presence (No-Auxin) of WAPL revealed that regions that are further than 0.5 Mb from the DSB site have significantly higher levels of RAD51 after WAPL degradation (Fig. 4f, g and Fig. S26). These findings suggest that elongated chromatin loops result in a broader homology search.

To further explore the potential role of loop-extruding cohesin in HR, we studied the impact of NIPBL depletion. NIPBL is a cohesin subunit that is required for loop extrusion but not for sister chromatid cohesion; consequently, NIPBL knock-down perturbs the function of cohesin loops without disrupting sister chromatid cohesion (43). We co-transfected our previously characterized (Fig. 3k) mES cells with an *I-SceI* expression vector together with *Nipbl, Rad51* or control (luciferase) siRNA and determined HR efficiency 72 h later (see Methods). As expected, no HR products were detected in the “donor-less” clone (Fig. S27). Depletion of NIPBL in cells containing the original Donor +3 HR reporter reduced HR efficiency by ~46% in comparison to control siRNA (Fig. 4h, i), suggesting that cohesin-mediated loop extrusion contributes to DSB-induced HR, even when the donor is located only ~3 kb from the DSB. The impact of NIPBL depletion was more pronounced in the Donor+441 and Donor+563 cell lines, where the HR donor is located far from the DSB site, showing ~74% reduction (Fig. 4i). Thus, at large DSB-donor distances, the dependence of HR on NIPBL becomes larger. In contrast to NIPBL, RAD51 depletion had impacts on HR that were independent of the DSB-donor distance, with reductions of 94% (original HR reporter), 97% (average over Donor+441 clones) and 96% (the Donor+563 clone; Fig. 4j). Finally, depletion of WAPL on a subset of clones showed an opposite trend in distance-dependent HR compared to NIPBL, with distal DSB-donor clones exhibiting higher, control siRNA-normalized HR levels compared to the Donor+3 clone (Fig. S28). Taken together, our data suggest that loop extruding cohesin promotes HR by facilitating the homology search. Of note, the dependence of HR on loop extrusion is more pronounced when the homologous donor is positioned at a greater distance from the DSB.

### A role for cohesin loop extrusion in the homology search.

Based on our data, we propose that loop-extruding cohesin facilitates the homology search via directed 1D scanning of the local TAD. In post-replicative cells, DNA breaks can be repaired by HR through the assembly of a RAD51 filament at each of the two resected DNA ends (44). This filament can then perform a local search on the sister chromatid, which is discontinuously tethered to the broken chromosome by pre-existing deposits of cohesive cohesin (43). If the HR donor were not within reach of this local search, possibly because of imperfect sister chromatid alignment, or if the sister chromatid were also broken at the same locus so that equal sister chromatid recombination is not possible, this initial local search would be unproductive. Cohesin recruitment to the break site could then facilitate extrusion of a chromatin loop anchored at the break, allowing the RAD51 filament to perform an efficient 1-D scan of the chromosome for a homologous donor (see Fig. 4k). Although our data cannot tell if the DSB-chromatin contacts observed are within the broken chromosome or with the sister chromatid, a related work showed that both contribute to the Hi-C stripes (45).

Our findings invite investigation about additional aspects of homology search. For example, is cohesin extruding a loop *in cis* on the broken chromosome or is it extruding the sister chromatid by the broken end *in trans*, directionally scanning one DNA molecule with respect to the other (**see** Fig. 4k)? Another open question is whether the two ends of the DSB remain tethered to one another during the homology search, or whether they each perform a separate individual search (compare top and bottom panel in Fig. 4k). In yeast, the Rad51 nucleoprotein filaments on both sides of the break were proposed to be held together during the homology search, suggesting a coupled scanning mechanism (26, 46). This model remains to be tested in mammalian cells. How dynamic is the search and the formation of break-anchored chromatin loops? It has been proposed that chromatin loops are transient and rare (47) and it is likely that the break-anchored loops formed during HR are also transient, effectively increasing the 1D mobility of the break, rather than stably positioning it at a defined genomic location. Moreover, besides the break-anchored chromatin loops, other features of 3D genome architecture such as pre-existing loops and TADs, are also likely to regulate the extent of the homology search, as proposed in a yeast study (39). Future works will be aimed at quantifying the relative contribution of break-anchored loops and pre-existing 3D genome architecture to the homology search. In addition, the role in homology search of factors other than cohesin remains to be investigated, for instance Rad54, which can drive 1D scanning of the Rad51 filament along short stretches of DNA in vitro (38, 48). Finally, it is worth noting that our Cas9 system can, in some cases, induce DSBs in both sister chromatids, abrogating the canonical mode of equal sister chromatid recombination. Based on our imaging-based calibration of multi-target CRISPR (27), we estimate that the majority of breaks are likely limited to one sister chromatid, but further studies are needed to confirm this and to determine how the presence of a DSB in the sister locus affects the homology search.

Previous work using AsiSI restriction enzymes to generate DSBs (21) also reported the formation of break-anchored chromatin loops, leading to the proposal that γH2AX spreads over Mb scale through cohesin-powered loop extrusion. Although our data showed that break-anchored chromatin loops become detectable only *after* the assembly of the γH2AX chromatin domain (Fig. 1), it remained possible that cohesin plays a role in γH2AX chromatin domain formation. Indeed, acute RAD21 degradation resulted in a reduced accumulation of γH2AX (Fig. S29), supporting a role for cohesin in propagating γH2AX. One possibility is that transient break-anchored loops might form during the early stages of repair (<1h) and contribute to γH2AX spreading, but might exist at levels undetectable in our Hi-C assays. Alternatively, cohesin might promote formation of the γH2AX domain through pre-existing chromatin loops, rather than break-anchored loops. This mechanism would enable the DSB to interact with the damaged TAD and help propagate γH2AX without the need for an anchoring mechanism at the DSB. In this regard, recent work showed that cohesin can rapidly bring together two genomic loci with no anchoring properties, provided the loci reside in the same TAD (49).

The homology search problem posits the question: how does a broken DNA find a correct HR template in the mammalian genome? A 3D exploration mechanism, e.g. via 3D diffusion, has been proposed to drive homology searches during alternative lengthening of telomeres repair of telomeric DSBs (50). However, in non-repetitive regions, a 3D search mechanism is likely to be slow and inefficient (51). Dimensionality reduction via 1D local scanning could significantly accelerate this process, for example through sliding of the recombinase-ssDNA filament along dsDNA (52). However, because the two sister chromatids are only loosely connected, such scanning would require a highly processive and fast motor. Our data suggests that cohesin can facilitate this long-range scanning via loop extrusion. In the bacterium *Caulobacter crescentus*, live-cell imaging measurements revealed a fast, directional movement of bacterial recombinase (RecA) filaments over the entire bacterial chromosome on the order of minutes (53). This movement required another SMC protein, RecN, and the authors suggested that RecN could actively drive the search via 1D scanning. In yeast, chromatin loops are formed during HR, promoting break-chromatin interactions and facilitating contact with an HR donor *in cis* (26). Our data suggests that cohesin also plays a role in the homology search in mammalian cells, likely by promoting repeated long-range scanning of RAD51-ssDNA filaments against the sister chromatid and against other intra-TAD loci *in cis*. Taken together, these findings point to a universal role for SMC proteins in driving a directed 1D scanning during the homology search, likely conserved across different domains of life.

## Supplementary Material

Supplementary materials

## Data Availability

All data associated with this study are present in the main text or the supplementary materials. ChIP-seq and Hi-C data have been uploaded to the Sequence Read Archive under BioProject accession PRJNA1214218. Analysis code is available on GitHub (https://github.com/AlbertoMarinG/HomSearch/).

## References

[1] Dixon JR , Topological domains in mammalian genomes identified by analysis of chromatin interactions. Nature 485, 376–380 (2012). 22495300 10.1038/nature11082 PMC3356448

[2] Rao Suhas S. P. , A 3D Map of the Human Genome at Kilobase Resolution Reveals Principles of Chromatin Looping. Cell 159, 1665–1680 (2014). 25497547 10.1016/j.cell.2014.11.021 PMC5635824

[3] Rao SSP , Cohesin Loss Eliminates All Loop Domains. Cell 171, 305–320.e324 (2017). 28985562 10.1016/j.cell.2017.09.026 PMC5846482

[4] Schwarzer W , Two independent modes of chromatin organization revealed by cohesin removal. Nature 551, 51–56 (2017). 29094699 10.1038/nature24281 PMC5687303

[5] Fudenberg G , Formation of Chromosomal Domains by Loop Extrusion. Cell Reports 15, 2038–2049 (2016). 27210764 10.1016/j.celrep.2016.04.085 PMC4889513

[6] Davidson IF , DNA loop extrusion by human cohesin. Science 366, 1338–1345 (2019). 31753851 10.1126/science.aaz3418

[7] Kim Y, Shi Z, Zhang H, Finkelstein IJ, Yu H, Human cohesin compacts DNA by loop extrusion. Science 366, 1345–1349 (2019). 31780627 10.1126/science.aaz4475 PMC7387118

[8] Pombo A, Dillon N, Three-dimensional genome architecture: players and mechanisms. Nature Reviews Molecular Cell Biology 16, 245–257 (2015). 25757416 10.1038/nrm3965

[9] Bonev B, Cavalli G, Organization and function of the 3D genome. Nature Reviews Genetics 17, 661–678 (2016). 10.1038/nrg.2016.112 27739532

[10] Krijger PHL, de Laat W, Regulation of disease-associated gene expression in the 3D genome. Nature Reviews Molecular Cell Biology 17, 771–782 (2016). 27826147 10.1038/nrm.2016.138

[11] Hafner A, Boettiger A, The spatial organization of transcriptional control. Nature Reviews Genetics 24, 53–68 (2023). 10.1038/s41576-022-00526-0 36104547

[12] Yu M, Ren B, The Three-Dimensional Organization of Mammalian Genomes. Annual Review of Cell and Developmental Biology 33, 265–289 (2017). 10.1146/annurev-cellbio-100616-060531 PMC5837811 28783961

[13] Yang JH, Hansen AS, Enhancer selectivity in space and time: from enhancer–promoter interactions to promoter activation. Nature Reviews Molecular Cell Biology 25, 574–591 (2024). 38413840 10.1038/s41580-024-00710-6 PMC11574175

[14] Pope BD , Topologically associating domains are stable units of replication-timing regulation. Nature 515, 402–405 (2014). 25409831 10.1038/nature13986 PMC4251741

[15] Emerson DJ , Cohesin-mediated loop anchors confine the locations of human replication origins. Nature 606, 812–819 (2022). 35676475 10.1038/s41586-022-04803-0 PMC9217744

[16] Zhang Y , The fundamental role of chromatin loop extrusion in physiological V(D)J recombination. Nature 573, 600–604 (2019). 31511698 10.1038/s41586-019-1547-y PMC6867615

[17] Zhang Y, Zhang X, Dai H-Q, Hu H, Alt FW, The role of chromatin loop extrusion in antibody diversification. Nature Reviews Immunology 22, 550–566 (2022). 10.1038/s41577-022-00679-3 PMC9376198 35169260

[18] Canela A , Genome Organization Drives Chromosome Fragility. Cell 170, 507–521.e518 (2017). 28735753 10.1016/j.cell.2017.06.034 PMC6133249

[19] Yang JH, Brandão HB, Hansen AS, DNA double-strand break end synapsis by DNA loop extrusion. Nature Communications 14, 1913 (2023). 10.1038/s41467-023-37583-w PMC10079674 37024496

[20] Collins PL , DNA double-strand breaks induce H2Ax phosphorylation domains in a contact-dependent manner. Nature Communications 11, 3158 (2020). 10.1038/s41467-020-16926-x PMC7308414 32572033

[21] Arnould C , Loop extrusion as a mechanism for formation of DNA damage repair foci. Nature 590, 660–665 (2021). 33597753 10.1038/s41586-021-03193-z PMC7116834

[22] de Luca KL , Genome-wide profiling of DNA repair proteins in single cells. Nature Communications 15, 9918 (2024). 10.1038/s41467-024-54159-4 PMC11582664 39572529

[23] Danovski G , Diffusion of activated ATM explains γH2AX and MDC1 spread beyond the DNA damage site. iScience 27, (2024). 10.1016/j.isci.2024.110826 PMC11416226 39310780

[24] Ström L, Lindroos HB, Shirahige K, Sjögren C, Postreplicative Recruitment of Cohesin to Double-Strand Breaks Is Required for DNA Repair. Molecular Cell 16, 1003–1015 (2004). 15610742 10.1016/j.molcel.2004.11.026

[25] Ünal E , DNA Damage Response Pathway Uses Histone Modification to Assemble a Double-Strand Break-Specific Cohesin Domain. Molecular Cell 16, 991–1002 (2004). 15610741 10.1016/j.molcel.2004.11.027

[26] Piazza A , Cohesin regulates homology search during recombinational DNA repair. Nature Cell Biology 23, 1176–1186 (2021). 34750581 10.1038/s41556-021-00783-x

[27] Zou RS , Massively parallel genomic perturbations with multi-target CRISPR interrogates Cas9 activity and DNA repair at endogenous sites. Nature Cell Biology 24, 1433–1444 (2022). 36064968 10.1038/s41556-022-00975-z PMC9481459

[28] Aymard F , Genome-wide mapping of long-range contacts unveils clustering of DNA double-strand breaks at damaged active genes. Nature Structural & Molecular Biology 24, 353–361 (2017). 10.1038/nsmb.3387 PMC5385132 28263325

[29] Natsume T, Kiyomitsu T, Saga Y, Kanemaki MT, Rapid Protein Depletion in Human Cells by Auxin-Inducible Degron Tagging with Short Homology Donors. Cell Reports 15, 210–218 (2016). 27052166 10.1016/j.celrep.2016.03.001

[30] Liu Y , Very fast CRISPR on demand. Science 368, 1265–1269 (2020). 32527834 10.1126/science.aay8204 PMC7608738

[31] Rogakou EP, Pilch DR, Orr AH, Ivanova VS, Bonner WM, DNA Double-stranded Breaks Induce Histone H2AX Phosphorylation on Serine 139*. Journal of Biological Chemistry 273, 5858–5868 (1998). 9488723 10.1074/jbc.273.10.5858

[32] Crane E , Condensin-driven remodelling of X chromosome topology during dosage compensation. Nature 523, 240–244 (2015). 26030525 10.1038/nature14450 PMC4498965

[33] Aymard F , Transcriptionally active chromatin recruits homologous recombination at DNA double-strand breaks. Nature Structural & Molecular Biology 21, 366–374 (2014). 10.1038/nsmb.2796 PMC4300393 24658350

[34] Dupré A , A forward chemical genetic screen reveals an inhibitor of the Mre11–Rad50–Nbs1 complex. Nature Chemical Biology 4, 119–125 (2008). 18176557 10.1038/nchembio.63 PMC3065498

[35] Zhou Y, Caron P, Legube G, Paull TT, Quantitation of DNA double-strand break resection intermediates in human cells. Nucleic Acids Research 42, e19–e19 (2014). 24362840 10.1093/nar/gkt1309 PMC3919611

[36] Carreira A , The BRC Repeats of BRCA2 Modulate the DNA-Binding Selectivity of RAD51. Cell 136, 1032–1043 (2009). 19303847 10.1016/j.cell.2009.02.019 PMC2669112

[37] Peritore M, Reusswig K-U, Bantele SCS, Straub T, Pfander B, Strand-specific ChIP-seq at DNA breaks distinguishes ssDNA versus dsDNA binding and refutes single-stranded nucleosomes. Molecular Cell 81, 1841–1853.e1844 (2021). 33651987 10.1016/j.molcel.2021.02.005

[38] Crickard JB, Moevus CJ, Kwon Y, Sung P, Greene EC, Rad54 Drives ATP Hydrolysis-Dependent DNA Sequence Alignment during Homologous Recombination. Cell 181, 1380–1394.e1318 (2020). 32502392 10.1016/j.cell.2020.04.056 PMC7418177

[39] Renkawitz J, Lademann Claudio A., Kalocsay M, Jentsch S, Monitoring Homology Search during DNA Double-Strand Break Repair In Vivo. Molecular Cell 50, 261–272 (2013). 23523370 10.1016/j.molcel.2013.02.020

[40] Chandramouly G , BRCA1 and CtIP suppress long-tract gene conversion between sister chromatids. Nature Communications 4, 2404 (2013). 10.1038/ncomms3404 PMC3838905 23994874

[41] Wutz G , Topologically associating domains and chromatin loops depend on cohesin and are regulated by CTCF, WAPL, and PDS5 proteins. The EMBO Journal 36, 3573–3599 (2017). 29217591 10.15252/embj.201798004 PMC5730888

[42] Yesbolatova A , The auxin-inducible degron 2 technology provides sharp degradation control in yeast, mammalian cells, and mice. Nature Communications 11, 5701 (2020). 10.1038/s41467-020-19532-z PMC7659001 33177522

[43] Mitter M , Conformation of sister chromatids in the replicated human genome. Nature 586, 139–144 (2020). 32968280 10.1038/s41586-020-2744-4 PMC7116725

[44] Scully R, Panday A, Elango R, Willis NA, DNA double-strand break repair-pathway choice in somatic mammalian cells. Nature Reviews Molecular Cell Biology 20, 698–714 (2019). 31263220 10.1038/s41580-019-0152-0 PMC7315405

[45] Teloni F , Cohesin guides homology search during DNA repair via loops and sister chromatid linkages. bioRxiv, 2025.2002.2010.637359 (2025). 10.1126/science.adw0566 41343653

[46] Phipps J , Cohesin complex oligomerization maintains end-tethering at DNA double-strand breaks. Nature Cell Biology 27, 118–129 (2025). 39482358 10.1038/s41556-024-01552-2 PMC11735392

[47] Gabriele M , Dynamics of CTCF- and cohesin-mediated chromatin looping revealed by live-cell imaging. Science 376, 496–501 (2022). 35420890 10.1126/science.abn6583 PMC9069445

[48] Heyer W-D, Li X, Rolfsmeier M, Zhang X-P, Rad54: the Swiss Army knife of homologous recombination? Nucleic Acids Research 34, 4115–4125 (2006). 16935872 10.1093/nar/gkl481 PMC1616967

[49] Lee J , Kinetic organization of the genome revealed by ultra-resolution, multiscale live imaging. bioRxiv, 2025.2003.2027.645817 (2025). 10.1126/science.adx2202 PMC12818391 40966347

[50] Cho Nam W., Dilley Robert L., Lampson Michael A., Greenberg Roger A., Interchromosomal Homology Searches Drive Directional ALT Telomere Movement and Synapsis. Cell 159, 108–121 (2014). 25259924 10.1016/j.cell.2014.08.030 PMC4177039

[51] Renkawitz J, Lademann CA, Jentsch S, Mechanisms and principles of homology search during recombination. Nature Reviews Molecular Cell Biology 15, 369–383 (2014). 24824069 10.1038/nrm3805

[52] Ragunathan K, Liu C, Ha T, RecA filament sliding on DNA facilitates homology search. Elife 1, e00067 (2012). 23240082 10.7554/eLife.00067 PMC3510455

[53] Chimthanawala A , SMC protein RecN drives RecA filament translocation for in vivo homology search. Proceedings of the National Academy of Sciences 119, e2209304119 (2022). 10.1073/pnas.2209304119 PMC9674259 36346847

