# Supplementary material for: Cohesin drives chromatin scanning during the RAD51-mediated homology search
Source: Science. Author manuscript; Available in PMC 2025 Dec 14. (PMC12701822; doi:10.1126/science.adw1928)
Supplement: Supplementary materials [file NIHMS2128181-supplement-Supplementary_materials.pdf]

Supplementary Materials

Cohesin drives chromatin scanning during the RAD51-mediated homology search.

Alberto Marin-Gonzalez[1,2*], Adam T. Rybczynski[1,3*], Namrata M. Nilavar[4*], Daniel Nguyen[4], Andrew G. Li[4], Violetta Karwacki-Neisius[1,2], Roger S. Zou[5], Franklin J. Avilés-Vázquez[1,6], Masato T. Kanemaki[7,8,9], Ralph Scully[4,#] and Taekjip Ha[1,2,6,#]

1. Howard Hughes Medical Institute and Program in Cellular and Molecular Medicine, Boston Children's Hospital, Boston, MA, USA
2. Department of Pediatrics, Harvard Medical School, Boston, MA, USA
3. Department of Biology, Johns Hopkins University, Baltimore, MD, USA
4. Department of Medicine and Cancer Research Institute, Beth Israel Deaconess Medical Center and Harvard Medical School, Boston, MA, USA
5. Department of Medicine, Massachusetts General Hospital, Boston, MA, USA
6. Department of Biophysics and Biophysical Chemistry, Johns Hopkins University, Baltimore, MD, USA
7. Department of Chromosome Science, National Institute of Genetics, Mishima, Japan
8. Graduate Institute for Advanced Studies, SOKENDAI, Mishima, Japan
9. Department of Biological Science, Graduate School of Science, The University of Tokyo, Tokyo, Japan

*These authors contributed equally to this work.
[#]corresponding authors: T.H. (taekjip.ha@childrens.harvard.edu), R. S. (rscully@bidmc.harvard.edu)

Materials and methods

Cell culture

Human cell lines were cultured at 37 °C under 5% CO_2. Human embryonic kidney 293T (HEK293T, ATCC CRL-3216) cells were cultured in Dulbecco's Modified Eagle's Medium (DMEM, Corning 10-013), supplemented with 10% FBS (Corning, 35-011), and 1x Antibiotic-antimycotic (AA, Gibco 15240062). Human colorectal carcinoma 116 (HCT116) with WAPL tagged for auxin inducible degradation (AID) were grown in McCoy's 5A Medium (Gibco 16600082), supplemented with 10% FBS and 1x AA. Culturing media with FBS and AA will be referred to as complete media.

Mouse embryonic stem (mES) cells were thawed onto mouse embryonic fibroblasts and maintained on gelatinsed plates in mES cell culture medium (DMEM with L-glutamine, 4.5g/L glucose and sodium pyruvate) 15% serum, 20 mM HEPES pH 7.6, 0.1mM beta-mercaptoethanol, 500 U/mL rLIF, 1x MEM NEAA, 2 mM L-glutamine, 100 U/mL penicillin, 100 μg/mL streptomycin, 0.001M sodium pyruvate).

Cells were regularly tested for mycoplasma infection using Myco-Alert assay (Lonza).

Electroporation of Cas9 RNP

Guide RNA sequences are in **Table S1**. 2 μL of 100 μM crRNA (IDT, Alt-R™ CRISPR-Cas9 crRNA) and 2 μL of 100 uM tracrRNA (IDT, 1072532) were mixed and incubated on a 95 °C heat block for 3 minutes, then cooled to room temperature on a benchtop for 5 minutes to enable annealing of the crRNA/tracrRNA. 3 μL of Cas9 enzyme (IDT, 1081058) at 10 μg/μL and 8 μL of dialysis buffer (20mM HEPES pH 7.5, 500mM KCl, 20% glycerol) were then added to the annealed crRNA/tracrRNA and were mixed. The mixture was incubated at room temperature for at least 20 minutes in order for the RNP to form. 73.8 μL of SF (HEK293T) or SE (HCT116) and 16.2 μL of supplement solution (Lonza V4XC-2024, V4XC-1024) were freshly mixed prior to nucleofection. For mES cells, 82 μL of P3 solution was mixed with 18 μL of supplement solution (Lonza, V4XP-3024).

Approximately 1.2 x 10^7 cells were harvested via trypsinization, spun down at 300 x *g* for 5 minutes, washed with 3 mL of PBS (Corning, 21-040-CV) and spun down again (same spin). All the PBS was then removed. HEK293T/HCT116 cells were resuspended in 90 μL of SF/SE + supplement mixture. mES cells were resuspended in 100 μL of P3 + supplement mixture. 15 μL of Cas9-tracr/crRNA RNP and 2 μL of 100 uM electroporation enhancer (IDT, 1075916) were then added to the cells. After gently mixing, 130 μL of cells-RNP mixture were transferred into a 100 μL nucleofection cuvette (Lonza). Electroporations were done in a 4D Nucleofector Unit (Lonza) using the following programs: CA-189 for HEK293T, EN-113 for HCT116 and CG-104 for mES cells. Cells were then transferred into 10 mL of complete media and immediately plated into 10 cm dishes precoated with collagen (1:100 collagen [Gibco A1048301] in PBS for HEK293T, HCT116) or gelatin (for mES). A no-electroporation control was included in every electroporation experiment by harvesting ~8 x 10^6 cells and plating them in a 10 cm dish. Cells were harvested by removing the complete media and scraping in DMEM only (with no FBS). Cells were harvested 3 hours after electroporation unless otherwise specified.

For time-course experiments, tracrRNA was annealed with a caged crRNA (Biosynthesis, custom order) using the same protocol as for regular crRNA. Four electroporations were performed as described above, cells were then pooled into 50 mL of complete media and plated into five 10 cm dishes. Cells were cultured at 37 °C, 5% CO_2 for ~12 hours prior to Cas9 activation. This was done by exposing four of the 10 cm dishes to (6x) flashlights that delivered 1.3 J/cm^2 of 365nm wavelength light for ~ 1 minute. Cells were then harvested at different time points after light-induced Cas9 activation: 15 minutes, 30 minutes, 1 hour and 3 hours. The fifth dish was not exposed to light (referred to as "no-light control" or "0 min time point").

For DNA-PKcs inhibition experiments, cells were incubated with inhibitor-containing complete media for 1 hour prior to the electroporation and were plated in media containing inhibitor after the nucleofection (NU7441, Selleck Chem S2638, 5 μM final concentration). For Mirin experiments, cells (not pre-exposed to the inhibitor) were plated in complete media containing inhibitor after the nucleofection (Selleck Chem, S8096, 50 μM final concentration).

For HCT-WAPL-AID cells, cells were nucleofected as described above using two electroporations per experiment. Cells were pooled after the electroporation, then split into two 10 cm plates, one with auxin-containing complete media, one with complete media. This ensures that electroporation efficiency was the same in auxin treated and untreated cells. Cells that were not electroporated were also plated on 10 cm plates containing complete medium with auxin or complete media with no auxin.

Rad21 siRNA treatment

The day before siRNA transfections, HEK293T cells were plated in five 6 well plates at ~30% confluency in 2.25 mL of DMEM with 10% FBS (no AA) and incubated at 37 °C, 5% CO_2 overnight. For each well, 6.25 μL of Rad21 siRNA at 100 μM (10 nM final) (ThermoFisher, #4392420, id s11727) or control siRNA (ThermoFisher # 4390843) were mixed with 125 μL of OptiMEM (Gibco #51985034); and 7.5 μL of RNAiMAX lipofectamine (ThermoFisher #13778150) was mixed with 125 μL of OptiMEM. OptiMEM-diluted siRNA was then mixed with OptiMEM-diluted lipofectamine, and the mixture was incubated for 10 minutes in order for liposomes to form. Cells were then transfected by adding 250 μL of lipofectamine-siRNA mixture to each 6 well plate. The following day, cells were expanded to 10 cm plates (for electroporation) and 6 well plates (for Western blot harvest) as needed. Electroporations were done 48 hours after siRNA transfections. siRNA-mediated Rad21 depletion was validated using Western Blot (**Fig. S30**).

Western Blot

HEK293T cells treated with Rad21 or Ctrl siRNA were harvested via trypsinization, spun down at room temperature, 200 x *g* for 3 minutes, and the supernatant was discarded. Cells were then washed with in 1 mL PBS (same spin). The supernatant was discarded and ice cold 1x RIPA (Cell Signaling 9806S) with 1 mM PMSF (PMSF stock 200 mM in isopropanol – Cell Signaling Technology 8553S) was added. Cells were sonicated with a probe sonicator (Qsonica Q125) at 4 C for 10 seconds at 30% power. Samples were spun down at 4 C, 21,000 x *g* for 20 minutes. The supernatant was taken off and stored in aliquots in Eppendorf tubes, flash frozen and stored at -80 °C.

BCA assay was run to determine protein concentration prior to starting the western blot, following the protocol for Pierce BCA Protein Assay Kit (23227) and imaging using a Tecan Spark plate imager. Once protein concentration of the sample was determined, 50 μg of total cell lysate was loaded into an Eppendorf tube, then 4x Laemmli buffer (Biorad 1610747) was added together with nuclease free water to a final volume of 28 μL. Samples were mixed and boiled on a 95 °C heat block for 5 minutes. After the sample was boiled, a gel (Biorad 45601023EDU) was loaded with ladder (Biorad 161-0374), followed by protein sample mixtures and run in 1x TGS (Biorad 1610772) for 30 minutes at 80 V, then 1.5 hours at 100 V. The gel was transferred to a PVDF membrane (Biorad 1620174) via Biorad Trans-Blot Turbo machine. First, the membrane and blotting paper (Biorad 1703932) were wetted with solution (10 mL methanol, 5 mL 10x Tris/Glycine buffer (Biorad 1610771), 35 mL water), then the protein gel was broken out of the plastic mold. Wet blotting paper, then wet membrane, then wet gel, then wet blotting paper were placed into a *sandwich*, and this *sandwich* was placed into the Trans-Blot Turbo machine chamber. The machine was run for 30 minutes to ensure complete transfer of protein from gel to membrane. Then, the membrane was washed with TBST (1x TBS (diluted from 10X Quality Biological 351-086-101), 0.1% Tween-20 (Promega H5151)) for 5 minutes at room temperature on a rocker. The membrane was blocked with 5% BSA/TBST (VWR 0332) for 1 hour at room temperature on a rocker. Primary antibody was diluted to 1:2000 for Rad21 (Abcam ab992) and 1:3000 for Beta-tubulin control (Thermo MA5-16308) in 5% BSA/TBST. Blocking solution was taken off and the membrane was incubated in primary antibody mixture at 4 C overnight on a rocker.

The next morning, the membrane was washed with 1 quick TBST wash, then three times for five minutes each with TBST on a rocker at room temperature. Secondary antibody was diluted to 1:2500 anti mouse HRP (Invitrogen A28177) and 1:2500 anti rabbit HRP (Invitrogen 31460) in 5% BSA/TBST. The membrane was incubated in secondary antibody for 1 hour at room temperature on a rocker with the membrane chamber wrapped in foil and protected from light. Excess secondary antibody was discarded, and the membrane was washed with 1 quick TBST wash, then three times for five minutes each in TBST on a rocker at room temperature. ECL solution was made based on manufacturer's guidelines (Thermo 34080), TBST wash buffer was discarded, and ECL solution was put onto the membrane. The membrane was placed into a chemiluminescent-compatible imager (Azure Biosystems 400) and imaged.

Cell cycle synchronization

HEK293T cells were synchronized in G1 and late S/G2 cell cycle phases using, respectively, a double and a single thymidine block protocol. Cells were plated in 10 cm plates (pre-coated with collagen) at low confluency and allowed to sit for at least 3 hours prior to initiation of synchronization. For the double thymidine block, thymidine (5 mM final) in DMEM complete medium was added to cells and incubated at 37 °C, 5% CO_2 for 18 hours, then washed twice with PBS. DMEM complete medium was then added to cells and incubated at 37 °C, 5% CO_2 for 9 hours. DMEM was then removed and thymidine-containing DMEM complete medium was then added a second time to cells and incubated at 37 °C, 5% CO_2 for 18 hours. At this point, cells were collected and electroporated with caged gRNA-Cas9 RNP as outlined above. Light was shone 15 hours after release of the block, corresponding

to G1-synchronized cells (with ~92% of cells in G1, see **Fig. S9**). For the single thymidine block, thymidine (5 mM final) in DMEM complete medium was added to cells and incubated at 37 °C, 5% CO_2 for 18 hours, then cells were collected and electroporated with caged gRNA-Cas9 RNP as outlined below. Light was shone 6 hours after release of the block, corresponding to late S/G2 synchronized cells (with ~80% of cells in late S/G2, see **Fig. S8**).

Cells were harvested and fixed 3 hours after shining light and pellets were split into two: one was used for Hi-C and the second was used for MRE11 ChIP-qPCR to confirm efficient generation of DSBs. A no-light control was included in every experiment and harvested at the same time as the damaged sample.

<u>Chromatin immunoprecipitation (ChIP)</u>

We followed the ChIP protocol published in (*1*). We used at least 3-4 x 10^6 cells per ChIP, which corresponds to ~2 ChIPs per electroporation. Cells were collected via scraping in 10 mL of DMEM with no FBS and were then transferred to a 15 mL falcon tube. Cells were then fixed in formaldehyde (methanol free, 1% final concentration) for 10 minutes with rotation at room temperature. Formaldehyde was then quenched by adding glycine at a final concentration of 140 mM and incubating for 3 minutes with rotation at room temperature. Cells were then spun at 1,200 x *g* for 3 minutes at 4 °C, washed with PBS and spun down (same spin). Pellets were then subjected to lysis (see below) or, alternatively, were flash-frozen and stored at -80 °C (pellets are stable at -80 °C for several months).

50 μL of Protein A or Protein G magnetic beads (ThermoFisher, #10002D, #10004D) were used per ChIP reaction (e.g., for four ChIPs, add 200 μL of beads). Beads were transferred to a 2 mL Eppendorf tube and were washed three times with 1 mL of BSA blocking buffer (BSA-BB, 0.5% BSA in PBS) using a magnet to retain the beads during each wash. Beads were then resuspended in 100 μL of BSA-BB. Antibody was then added to the beads according to **Table S2**. The antibody was allowed to bind to the magnetic beads at room temperature with rotation for 1 to 3 hours. Right before the immunoprecipitation, beads were washed three times with 1 mL BSA-BB and resuspended in 50 μL of BSA-BB per ChIP reaction.

Cell lysis was done in three steps. First, cell pellets were resuspended in 4 mL of LB1 lysis buffer (50 mM HEPES, 140 mM NaCl, 1 mM EDTA, 10% glycerol, 0.5% Igepal CA-630, 0.25% Triton-X; with pH adjusted to 7.5 using KOH and with 1x protease inhibitors [ThermoFisher, 78429] added right before use) and incubated for 10 min with rotation at 4 °C. Cells were then spun down for 3 min at 2,000 x *g* and 4 °C. The supernatant was removed, and cells were resuspended in 4 mL of LB2 buffer (10 mM Tris-HCl pH 8, 200 mM NaCl, 1 M EDTA and 0.5 mM EGTA; with pH adjusted to 8 using HCl and with 1x protease inhibitors added right before use) and incubated for 5 minutes with rotation at 4C. Cells were then spun down (3 min, 2,000 x *g* at 4 °C) and the supernatant was removed. Cells were then resuspended in 1.5 mL of LB3 buffer (10 mM Tris-HCl pH 8, 100 mM NaCl, 1 mM EDTA, 0.5 mM EGTA, 0.1% Na-Deoxycholate, 0.5% N-lauroylsarcosine; with pH adjusted to 8.0 using HCl, and adding 1x protease inhibitor right before use) and were transferred to a 2 mL tube. Cells were sonicated at 4 °C using a Qsonica Q125 with the following settings: 30 seconds of pulse, 30 seconds off, 55% amplitude for 6 cycles. Cells were then spun at 20,000 x *g* at 4 °C for 10 minutes. A small black pellet should appear at the bottom of the tube. ~13 μL (1% input chromatin) of supernatant was transferred to a 1.5 mL tube and reverse crosslinked and purified as the ChIP'ed DNA (described below). The rest of supernatant was transferred to a 5 mL tube (split into two tubes at this point when performing two ChIPs on one pellet), avoiding carrying any pellet. To the supernatant containing sonicated chromatin, we then added 300 μL of 10% Triton X-100, 50 μL of antibody-conjugated beads and LB3 (with no protease inhibitor) to a volume of 3 mL. The mixture was incubated for 7+ hours at 4 °C with rotation.

Samples were transferred to a 1.5 mL LoBind Eppendorf tube placed on a magnetic stand (removing the supernatant as it became cleared and adding more bead slurry). The supernatant was fully removed, and the beads were gently washed six times with 1 mL RIPA buffer (50 mM HEPES, 500 mM LiCl, 1 mM EDTA, 1 % Igepal CA-630, 0.7 % Na-Deoxycholate; pH adjusted to 7.5 using KOH) and once with TBS buffer (20 mM Tris-HCl pH 7.5, 150 mM NaCl). Beads were then resuspended in 50 μL of ChIP elution buffer (50 mM Tris-HCl pH 8, 10 mM EDTA, 1% SDS) and incubated at 65 °C for 7+ hours with shaking at 1,000 rpm.

For purification of the ChIP'ed DNA, 25 μL of IDTE buffer pH 8 (IDT) was added to the samples to dilute the SDS, followed by 2 μL of RNAse A (NEB), mix and incubation at 37 °C for 15 minutes. 4 μL of Proteinase-K (NEB) was then added and mixed, and samples were incubated at 55 °C for 1 hour. DNA was then purified using MinElute DNA purification columns (Qiagen) and eluted in 35 μL of IDTE pH 8.

ChIP-Seq sequencing libraries were prepared using the NEB Ultra II DNA Library Prep Kit (NEB, #E7645) following the standard protocol provided, using half volumes of library prep reagents and using 15 μL of purified ChIP DNA as input. After adapter ligation and USER treatment, libraries were amplified using NEBNext® dual-indexed primers (NEB 7600 or NEB 6440) following the provided protocol but using half volumes. Libraries were amplified using 13 PCR cycles (except for γH2AX ChIP samples where we used 10 cycles). Samples were quantified via Qubit (Thermo), pooled according to their concentration and sequenced on an Illumina NextSeq 1000 machine using paired-end 2x60 bp read length. Reads were demultiplexed using bcl2fastq.

qPCR

qPCR was done using the Luna Universal qPCR Master Mix (NEB, M3003) following the NEB protocol, using 1 μL of ChIP'ed DNA per reaction and the following primers: AluGG-s2-3 forward: 5'-TGGTGTACACTCCTTCCAGA, AluGG-s2-3 reverse: 5'-TTTTCACATGCTTGCTTCACA, AluGG-s3-3 forward: 5'- CAGTGGAGGAATTGGCCCA, AluGG-s3-3 reverse: 5'- CACTCTACCTGGCCCTTCC, AluGG-s4-4 forward: 5'- TCTCTACTTCCCTATATGGCCAC, AluGG-s4-4 reverse: 5'- GTGCATGGCCACCTTTAGT.

Strand-specific ChIP-Seq

RAD51 ChIP was carried out as described previously (*2*) with minor modifications. Briefly, cells were lysed in lysis buffer (50 mM Tris-HCl pH 8.0, 0.1% SDS, 10 mM EDTA supplemented with proteinase inhibitor) after formaldehyde crosslinking. Chromatin was sonicated to ~500 bp using Bioruptor 300 (Diagenode). Lysates from 6 million cells were used for each IP with anti-RAD51 antibody (Millipore, see **Table S2**). Immunocomplexes were captured by Magna ChIP magnetic beads (Sigma). Beads were washed six times with ChIP RIPA buffer (50 mM HEPES pH 7.6, 1 mM EDTA, 0.7% sodium deoxycholate, 1% NP-40) followed by two washes with TE buffer. DNA was eluted in ChIP elution buffer (1% SDS, 100 mM sodium bicarbonate, 8 μg/mL RNase A). After reverse crosslinking, DNA was precipitated with ethanol after phenol:chloroform extraction.

Strand-specific ChIP-seq libraries were prepared using the xGen ssDNA & Low-Input DNA kit with xGen CDI primers (IDT) according to the manufacturer's protocol.

Hi-C

Hi-C was done following the in-situ Hi-C protocol from (*3*) with some modifications. 2-4 x 10^6 cells were used per Hi-C reaction. Cells were harvested in 4 mL of DMEM only (no FBS) and fresh formaldehyde was added to a final concentration of 1%. The fixation reaction incubated for 10 minutes at room temperature and was quenched by adding glycine to a final concentration of 0.2 M and incubating for 5 minutes at room temperature with rotation.

Cells were then spun down (1,200 x *g*, 3 minutes, 4 °C), washed with 1 mL of PBS and spun down (same spin). PBS was removed and the pellets were subjected to lysis or frozen and stored at -80 C for later use (pellets are stable for several months at -80 °C).

Cell pellets were gently resuspended in 300 μL of lysis buffer (10 mM Tris-HCl pH 8, 10 mM NaCl, 0.2% Igepal) with 1x protease inhibitors (Sigma, P8340). The lysis reaction incubated on ice for ~20 minutes. After that, the sample was centrifuged (2,500 x *g*, 5 minutes, 4 °C), the supernatant was discarded, and nuclei were washed with 500 μL of ice-cold lysis buffer (with no protease inhibitors) and spun down (same spin). Nuclei pellets were then gently resuspended in 50 μL of 0.5% SDS and incubated at 62 °C for 10 minutes with shaking at 900 rpm. 145 μL of H_2O and 25 μL of 10% Triton X-100 (in H_2O) were added, and the sample was gently mixed and incubated at 37 °C for 15 minutes with shaking at 900 rpm. 25 μL of 10xNEBuffer-3.1 and 100 U of DpnII enzyme were added to the sample and gently mixed and chromatin was digested at 37 °C overnight with shaking at 900 rpm. The following day, an additional 100 U of DpnII enzyme were added to the sample and the digestion was incubated for another ~3-4 hours. DpnII was deactivated by incubating at 62 °C for 20 minutes and the sample was then left to cool down to room temperature. A fill-in reaction mix was then prepared in order to blunt the DNA ends and incorporate a biotin (30 μL of 0.4 mM biotin-14-dATP (ActiveMotif 14139), 1.2 μL of 10 mM dCTP (NEB), 1.2 μL of 10 mM dGTP (NEB), 1.2 μL of 10 mM dTTP (NEB) and 6.4 μL of Large Klenow Fragment at 5 U/μL (NEB, M0210)) and was added to the sample. After mixing, the fill-in reaction was incubated at 37 °C for 45 minutes with shaking at 900 rpm. A ligation reaction mix was then prepared (669 μL of H2O, 120 μL of 10xNEB T4 DNA ligase buffer, 100 μL of 10% Triton X-100 and 6 μL of 20 mg/mL BSA) and was then added to the sample. 5 μL of T4 DNA ligase was added, and the sample was mixed well and incubated at room temperature for >4 hours with rotation. After ligation, the sample was spun down (2,500 x *g*, 5 minutes, room temperature) resulting in a nuclei pellet, the supernatant was removed and the pellet was resuspended in 330 μL of reverse crosslinks buffer (10 mM Tris-HCl pH 8, 0.5 M NaCl, 1% SDS). After adding 20 μL of Proteinase-K (NEB), samples were incubated at 55 °C for 30 minutes and then at 68 °C overnight with shaking at 1000 rpm. The following day, DNA was purified using EtOH precipitation and was eluted in 130 μL of 10 mM Tris-HCl pH 8.

The DNA was transferred to a Covaris microTUBE (Covaris, 520045) and sonicated in a Covaris LE220 instrument using the following parameters: Fill Level: 10, Duty Cycle: 15, PIP: 500, Cycles/Burst: 200, Time: 58 seconds. Sonicated DNA was transferred to a 1.5 mL LoBind tube and subjected to double-sided size selection using AMPure XP beads (Beckman A63881), using 0.55x of beads for first round and 0.7x for the second round of selection (results in fragments between ~300 bp and 500 bp). After the second round, DNA was eluted in 100 μL of 10 mM Tris-HCl pH 8, separated on a magnet and transferred to a clean 1.5 mL LoBind tube. Size-selected DNA was then quantified with Qubit (ThermoFisher). DNA can be stored at -20 °C for up to three days at this point.

For biotin pull-down and library preparation, 2 μg of DNA was transferred into a clean 1.5 mL LoBind tube and 10 mM Tris-HCl pH 8 was added to a final volume of 100 μL. 100 μL of streptavidin beads (Life tech, 65602) were added to a clean 1.5 mL tube and were washed with 100 μL of Tween Washing Buffer (TWB: 5 mM Tris/0.5 mM EDTA, 1M NaCl, 0.05% Tween-20). Beads were placed against a magnetic stand, the supernatant was discarded, and beads were then resuspended in 100 μL of 2x Binding Buffer (10 mM Tris-HCl pH 7.5, 1 mM EDTA and 2 M NaCl). Beads were then mixed with the samples and the mixture was incubated at room temperature for ~20 minutes. Samples were then placed on a magnetic stand, the supernatant was removed and the beads containing biotinylated DNA fragments were washed twice by resuspending in 200 μL of TWB, incubating at 55 °C for 2 minutes and placing against the magnet. After the second wash, beads were resuspended in 100 μL of 10 mM

Tris-HCl pH 8, then placed against the magnet, removed the supernatant and resuspended in 50 μL of 10 mM Tris-HCl pH 8 and transferred to a PCR tube.

Library preparation was done on the beads, using the NEB Ultra II DNA Library Prep Kit (NEB, #E7645) with small variations from the NEB protocol to account for the beads. 7 μL of End Prep Rxn Buffer and 3 μL of End Prep Enzyme Mix were added to 50 μL of beads-DNA mixture, the sample was mixed and incubated: 20 °C for 30 minutes, then 60 °C for 30 minutes in a thermocycler. A ligation mix was then prepared (30 μL of NEB Ligation MM, 1 μL of ligation enhancer and 2.5 μL of undiluted Illumina Adapter), added to the sample, mixed and incubated at 20 °C for 15 minutes in a thermocycler. After adding 3 μL of USER enzyme and mixing, samples were incubated at 37 °C for 15 minutes. Samples were then washed twice with 150 μL of TWB (as described above), resuspended in 100 μL of IDTE pH 8, placed against a magnet, the supernatant was removed, and samples were finally eluted in 15 μL of IDTE pH 8. Samples were then incubated at 98 °C for 10 minutes on a thermocycler with lid heated to 105 °C, in order for the DNA to detach from the beads. Shortly after this incubation (let ~1 minute for tubes to cool down), samples were placed against a magnet and the supernatant was transferred to a clean 1.5 mL DNA LoBind tube.

Libraries were amplified using NEBNext® dual-indexed primers (NEB 7600 or NEB 6440) following the NEB protocol. 10 PCR cycles were run for each sample with annealing temperature of 65 °C. Amplified libraries were purified using two rounds of 0.9x AMPure XP bead purification and eluted in 25 μL of IDTE. Samples were quantified via Qubit (Thermo), pooled according to their concentration and sequenced on an Illumina NextSeq 1000 or a NovaSeq 6000 machine using paired-end 2x60 bp read length. Reads were demultiplexed using the bcl2fastq software.

<u>ChIP-Seq data analysis</u>

ChIP-Seq fastq reads were aligned to the hg38 (human cell lines) or the mm10 (mouse) genome assembly using bowtie2. For Rad51 ChIP-Seq experiments on the donor+441, donor+563 or donor-less cells, reads were aligned to a modified version of the mm10 genome assembly containing the HR reporter as specified. The samtools software was used to filter reads by mapping quality (>=25), remove singletons, remove PCR duplicates and index bam files (see github link accompanying the manuscript).

Average ChIP-Seq profiles were obtained using a custom python code that computes a rolling average around every on-target site for a given multi-target gRNA. For 3 Mb-wide profiles, the rolling average was computed every 5 kb, using a bin size of 10 kb; for 30 kb-wide profiles, the rolling average step was 50 bp and the bin size was 100 bp. Mean on-target profiles were then computed by averaging over all the on-target sites for the damaged and undamaged samples. Finally, the averaged undamaged profile was subtracted from the averaged damaged one, obtaining an enrichment profile. Enrichment profiles were averaged over biological replicates (Fig. 1e, f and Fig. 3a-d), or shown separately for each replicate (Fig. 1g, h, Fig. 2m, n and Fig. f, g). ChIP-Seq profiles at single cut sites were obtained by 1) subtracting the untreated from the Cas9-treated bam files and converting to a bigwig file (using the bamCompare script from deeptools {Ramírez, 2014 #1009} with default parameters); and 2) binning and plotting using the software pygenometracks {Lopez-Delisle, 2021 #1010}. 33 kb bins were used for coarse plots (e.g. **Fig. 4c**) and 1 kb bins were used for fine plots (e.g., **Fig. 3l**).

ChIP-Seq spread widths for RPA and RAD51 (Fig. 3e) were computed after merging biological replicate datasets. Narrow widths (columns #1, 2, 4 in Fig. 3e) were computed using the MACS2 software with the following parameters *--broad --broad-cutoff 0.05 --max-gap 5000 --min-length 500*. Wide widths (columns #3, 5) were calculated using a previously written python code (*1*). In short, a rolling average of ChIP-Seq signals are computed

for the damaged and undamaged sample in bins of 10 kb separated by steps of 5 kb. This rolling average is the compared in the damaged and undamaged sample at increasing distances from the DSB site. A counter is increased every time the damaged signal is lower than the undamaged one. The spread width is defined as the distance at which the counter reaches 10. RAD51 spreads at single cut sites (ACTB and MYC) were obtained using MACS2 with the following parameters *--broad --broad-cutoff 0.1 --max-gap 200,000 --min-length 5,000*.

TAD boundaries available from previous high-resolution Hi-C maps (*3*) (4DNFIBKY9EG9 file in 4DN portal) were used to obtain RAD51 enrichment across TAD boundaries (Fig. 4a). Only "strong" TAD boundaries (according to the labels in the original bed file) were considered for the analysis. RAD51 ChIP-Seq signal was extracted around TAD boundaries that lie at distances between 200 kb and 700 kb from the 100 most highly efficiently cleaved AluGG cut sites (as given by BLISS signal). This resulted in 155 TAD boundaries being analyzed. Rolling average profiles (bin size = 20 kb, step size = 10 kb, total span = 300 kb) were computed around each TAD boundary for the damaged and the undamaged sample. A normalized average around all TAD boundaries was computed for the damaged and undamaged sample by dividing each individual profile by the total RAD51 signal in the damaged sample and then computing the average over the normalized profiles. The resulting averaged normalized undamaged profile was subtracted from the damaged profile and is shown in Fig. 4A. In Fig. 4B, the change in RAD51 signal at each TAD boundary/random site was quantified and normalized by the total RAD51 signal in the damaged sample.

Strand-specific ChIP-Seq analysis

Strand specific ChIP-Seq profiles were obtained in the same manner as regular ChIP-Seq profiles. In addition, a custom python code was written to classify mapped paired-end reads attending to whether the first read mapped to the forward or the reverse strand and split accordingly. Enrichment profiles around AluGG sites were computed for the forward and reverse strand and the latter was subtracted from the former to obtain the strand asymmetry plots.

Hi-C analysis

Deep Hi-C contact maps for damaged and undamaged cells (Fig. 1a) were obtained using the *cooler* software (to obtain pairs file, then sort, remove duplicates, select only appropriately oriented pairs and merge technical and biological replicates) and *juicer* (to obtain hic files from the final pairs file). All other Hi-C contact maps were obtained with juicer (to go from .fastq to .hic) using default parameters. JuiceBox {Durand, 2016 #1011} was used for visualization of Hi-C contact maps (Fig .1a). Average log2 ratio plots (as in e.g., **Fig. 1b**) were obtained from the hic file of the damaged and undamaged sample using a custom python script. For each cut site, Hi-C matrices are dumped (using juicer, spanning 3 Mb, resolution = 50 kb, KR normalization) for the undamaged and damaged sample and the log2 ratio matrix is computed. The average of all these cut-centered log2 ratio matrices is then computed and shown. Bins with no contact count in the denominator were taken as zero in the log2 ratio.

Insulation scores were computed from .hic files in three steps: 1) dumping whole-chromosome matrices at 25 kb resolution from the hic files using juicer, 2) using a custom bash code to convert juicer matrices to txt suitable for cworld input, and 3) using the matrix2insulation.py function of the cworld package to extract the insulation score profile across the chromosome (see bash file in associated github site). A custom python code was then used to compute the insulation profiles around the AluGG on-target sites for the damaged and undamaged sample (similar to the ChIP-Seq profiles). Averaged insulation scores profiles around all on-target sites were obtained for each biological replicate and then averaged between replicates (e.g. **Fig. 2a**).

4C-like plots were computed from the merged Hi-C contact map obtained in untreated HEK293T using the hicPlotViewpoint function of HiCExplorer package using the cut site as viewpoint and a resolution of 25 kb.

mES cell line generation

mES cells were thawed onto irradiated mouse embryonic fibroblasts and maintained on gelatinzed plates in mES cell culture medium and were regularly tested for mycoplasma infection using Myco-Alert assay (Lonza). The founder mES cell lines contain Brca1$^{fl/exon11}$ alleles (*4*) along with a single copy of a DSB-inducible HR reporter targeted to the *Rosa26* locus. This reporter contains a unique 18 bp target site for the homing endonuclease I-SceI. Using CRISPR Cas9-mediated deletion, we removed the Δ5' *GFP* copy from the original reporter clone to generate a "donor-less" clone (Clone #8), which is incapable of generating GFP+ recombinant products. This clone was then subjected to CRISPR Cas9-assisted targeting of a new Δ5'-*GFP* donor sequence 441kb or 563kb from the *I-SceI* site using target-specific sgRNA. The new Δ5'-*GFP* donor contained silent mutations in the *GFP* and polyadenylation signal sequences that enable it to be distinguished from *GFP-I-SceI* during sequence alignment. Full details of this reporter, which also contains a red fluorescent protein expression cassette for detection of long tract gene conversion, will be described elsewhere. The clones were analyzed by PCR to confirm homologous insertion of both 5' and 3' targeting arms of the Δ5'-*GFP* donor. They were further analyzed by Southern blotting to confirm copy number and intactness of the Δ5'-*GFP* donor. Conventional Southern blotting, using gDNA digested with local restriction endonucleases flanking the site of insertion and with use of a *GFP* probe, was used to confirm single copy integration of the Δ5'-*GFP* donor. To distinguish between Δ5'-*GFP* donor targeting to the same chromosome as *GFP-I-SceI vs*. targeting to the homolog, we made use of a restriction site for the rare-cutting enzyme I-CeuI, which we included in the outer (telomeric) targeting arm of the Δ5'-*GFP* donor targeting vector. Digestion of gDNA agarose plugs with I-CeuI + I-SceI would yield *GFP*-hybridizing bands that migrate at ~443 kb (Donor+441 clones) or ~565 kb (Donor+563 clones), only if the Δ5'-*GFP* donor is positioned on the same chromosome as *GFP-I-SceI*. We identified four clones each of Donor+441 and Donor +563, in which a single, intact copy of the Δ5'-*GFP* donor was correctly targeted to the same chromosome as *GFP-I-SceI*. Clones in which the Δ5'-*GFP* donor was targeted to the homolog were not included in the current study.

Southern blotting

gDNA was isolated from clones using Puregene DNA isolation kit (Qiagen). 5 μg of gDNA was digested with appropriate restriction enzymes at 37 °C and run on 0.8% agarose in 0.5X TBE gel overnight. After denaturation, DNA was transferred onto a positively charged nylon membrane and probed with a ^{32}P-labelled *GFP* cDNA probe. Signal was recorded on a phosphorimager screen and scanned using a Typhoon biomolecular imager.

Pulsed field gel electrophoresis

0.8% agarose plugs were made using Clean cut agarose using $1x10^6$ cells. Plugs were then subjected to Proteinase K treatment overnight at 50 °C followed by three washes at 4 °C. Prior to the last wash, the plugs were treated for 1 hour with RNase A. One half of the plug was then washed with 10mM and 0.1mM EDTA for 1h and then equilibrated in 1xCutSmart buffer for 1 hour at 4 °C. The buffer was replaced with reaction containing 30 U of I-SceI and I-CeuI with 1xCutSmart buffer. The reaction was then incubated overnight in a thermomixer at 37 °C with gentle agitation at 300rpm. Next day, the plugs were washed with TE buffer for 30 minutes and then with 0.5X TBE for another 30 minutes. The plugs were then placed in the wells of an 1% agarose gel and electrophoresed for 24 hours at 4.5 V, 90-180 switch time, at 14 °C. The gel was then subjected to Southern blotting, probed with a ^{32}P-labelled *GFP* cDNA probe. Signals were detected by use of phosphorimaging, as described above.

Recombination assays

200 μL of 0.8 x 10^6 cells/mL were transfected in suspension in 24-well plates with 0.5 μg of pcDNA3β-myc NLS-I-SceI or 0.5 μg control empty vector, using Lipofectamine 2000 (Invitrogen). GFP^+ frequencies were measured 3 days post transfection using Beckman Coulter CytoFlex LX. Transfection efficiency was measured simultaneously by parallel transfection with 0.05 μg *GFP* expression vector with 0.45 μg empty vector. For siRNA experiments, cells were transfected with 20 pmol siRNA with 0.3 μg of pcDNA3β-myc NLS-I-SceI (or empty vector) per well. Repair frequencies were corrected for background events and for transfection efficiency (50–90%). HR data represent the mean and standard error of the mean of three or five independent experiments. Statistical analysis was by two-tailed paired *t*-test (unknown variance). siRNA efficiency was quantified by means of RT-qPCR (**Fig. S31**).

Supplementary figures and captions.

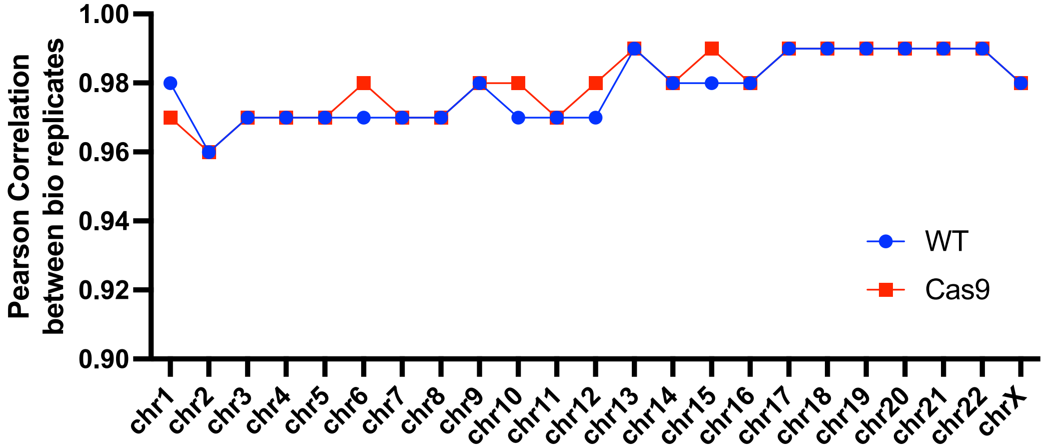

Fig S1. Hi-C reproducibility analysis. Pearson correlation coefficient was computed to compare contact frequencies between biological replicates in each chromosome. Chromosome contacts were obtained in windows of 25 kb.

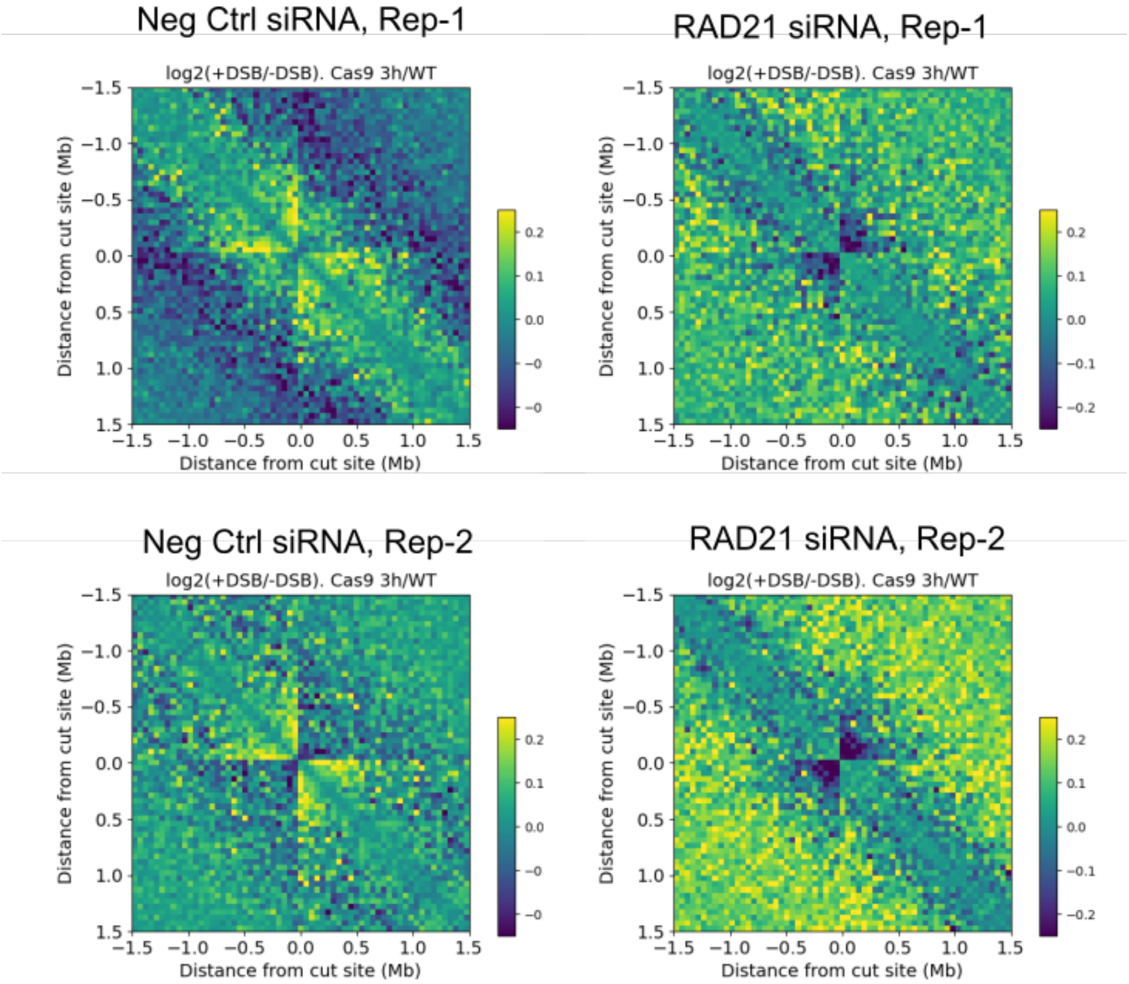

Fig. S2. Cas9-induced changes in Hi-C contacts in HEK293T upon treatment with control (left) or RAD21 (right) siRNA. Biological replicates are shown in separate rows. Analysis and plot details are the same as in Fig. 1d. Hi-C was done 3h after RNP nucleofection, which in turn was done 48h after siRNA treatment.

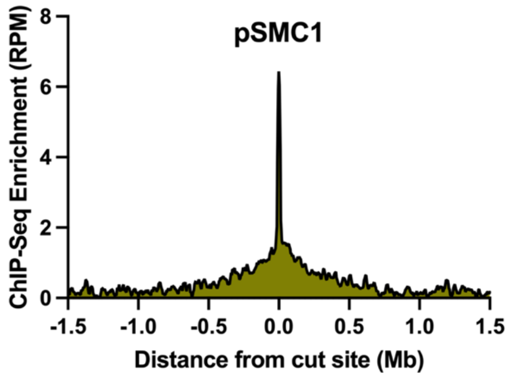

Fig. S3. ChIP-Seq profile for phosphorylated SMC1 (pSMC1) cohesin subunit around Cas9 on-target sites. Analysis and plot details are the same as in Fig. 1 f, e, main text.

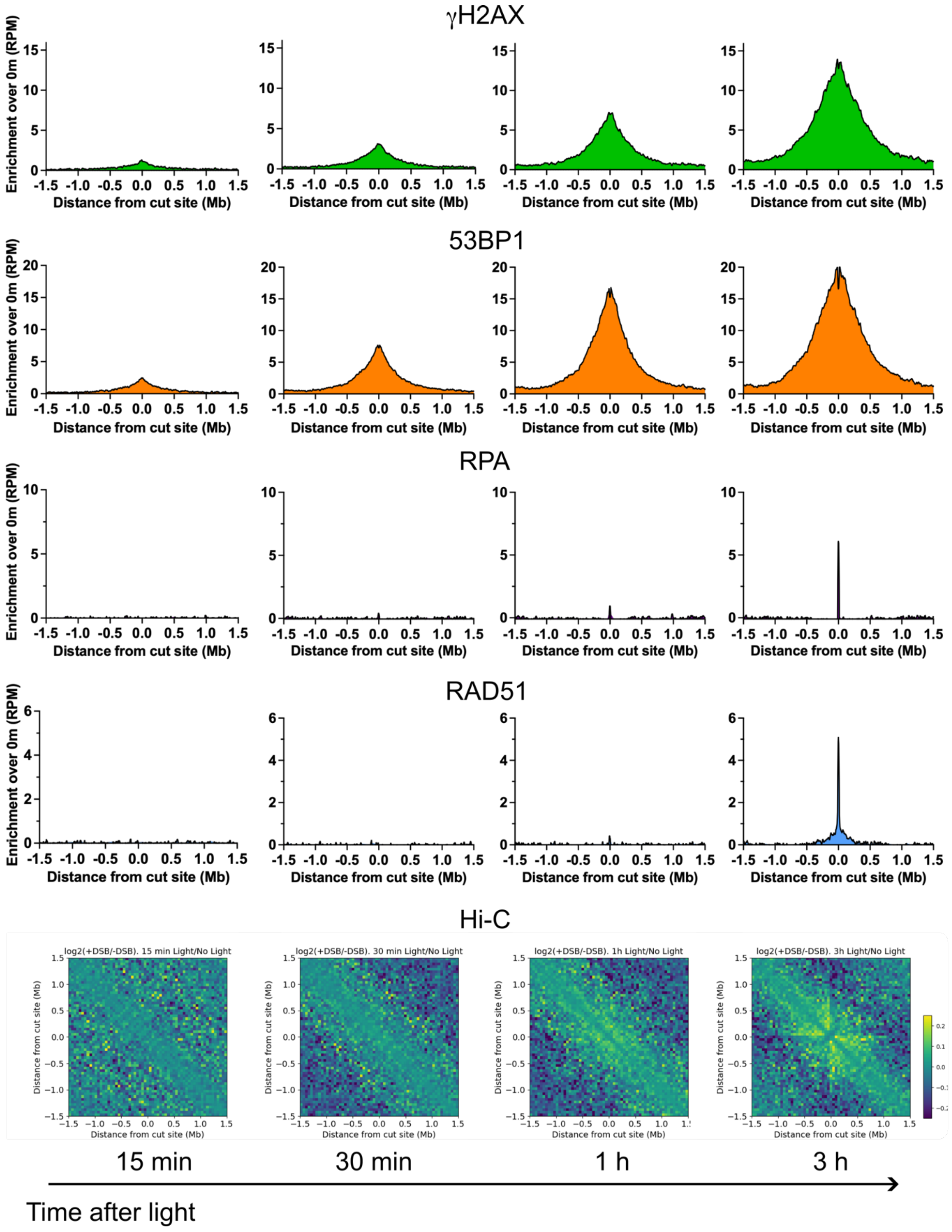

Fig. S4. Time-course ChIP-Seq and Hi-C, second replicate. Biological replicate of Fig. 1g-i and Fig. 2 m-o, main text. Data analysis and plot details are the same as in **Fig. 1g-i** and **Fig. 2 m-o**.

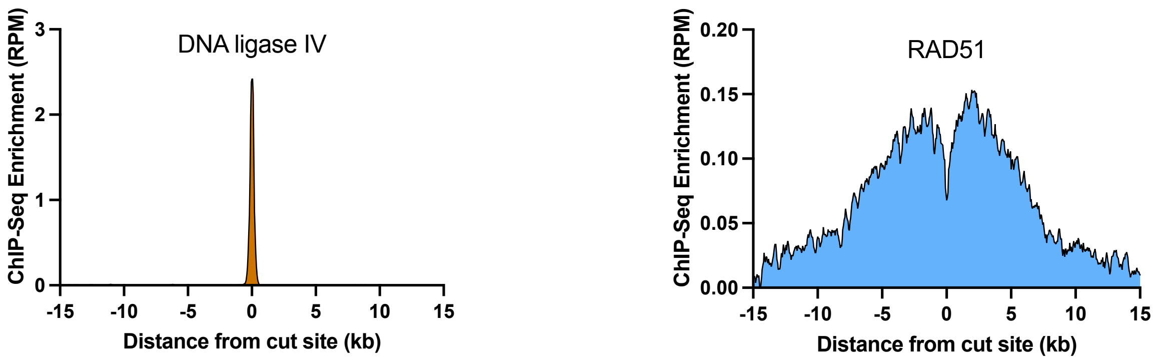

Fig. S5. ChIP-Seq profiles for DNA ligase IV and RAD51 around Cas9 cut sites. Analysis and plot details are the same as in Fig. 1 f, e, main text.

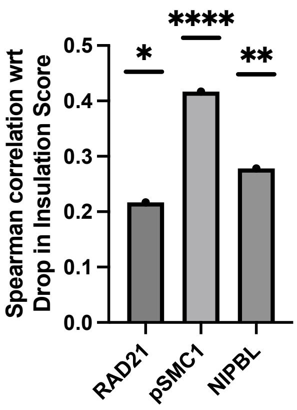

Fig. S6. Spearman correlations between DSB-induced cohesin enrichment and drop in insulation score. Analysis and plot details are the same as in Fig. 2e, main text.

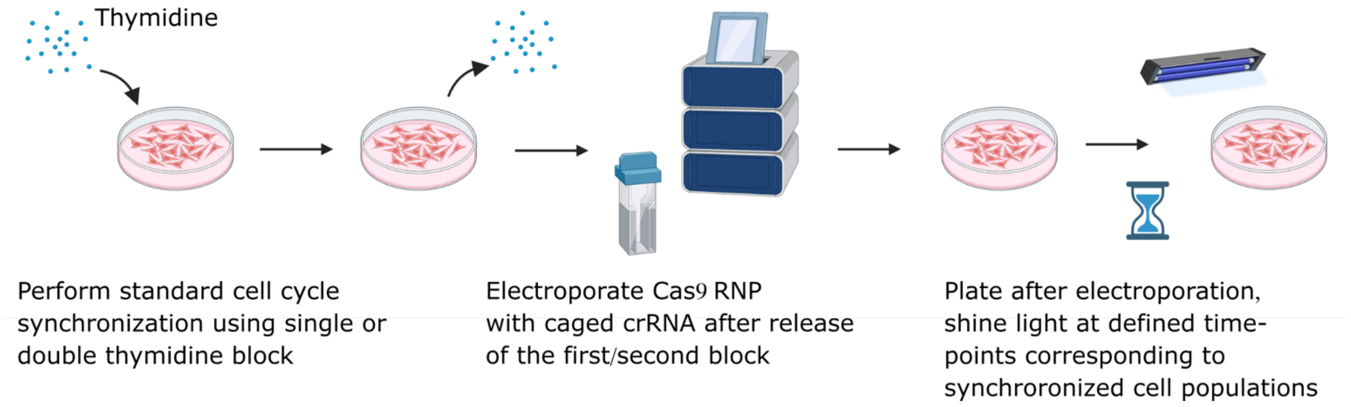

Fig. S7. Cartoon summarizing the cell cycle synchronization + vfCRISPR protocol to induce DSBs in synchronized cell populations.

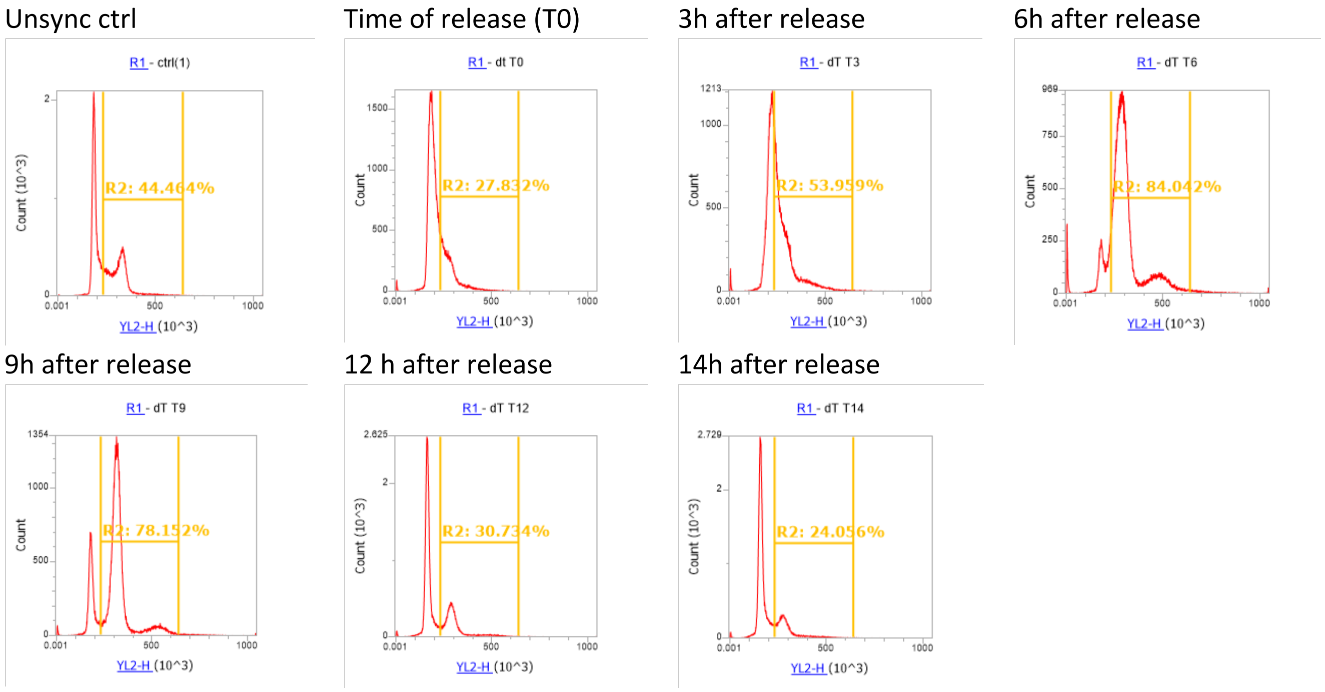

Fig. S8. FACS time-course measuring DNA content in HEK293T after release of first thymidine block. Cell cycle synchronization was performed using a single thymidine block (see methods). Cells were electroporated after release of the block, plated in 10 cm dishes and were harvested at different time points. DNA content was measured in flow cytometry via PI staining.

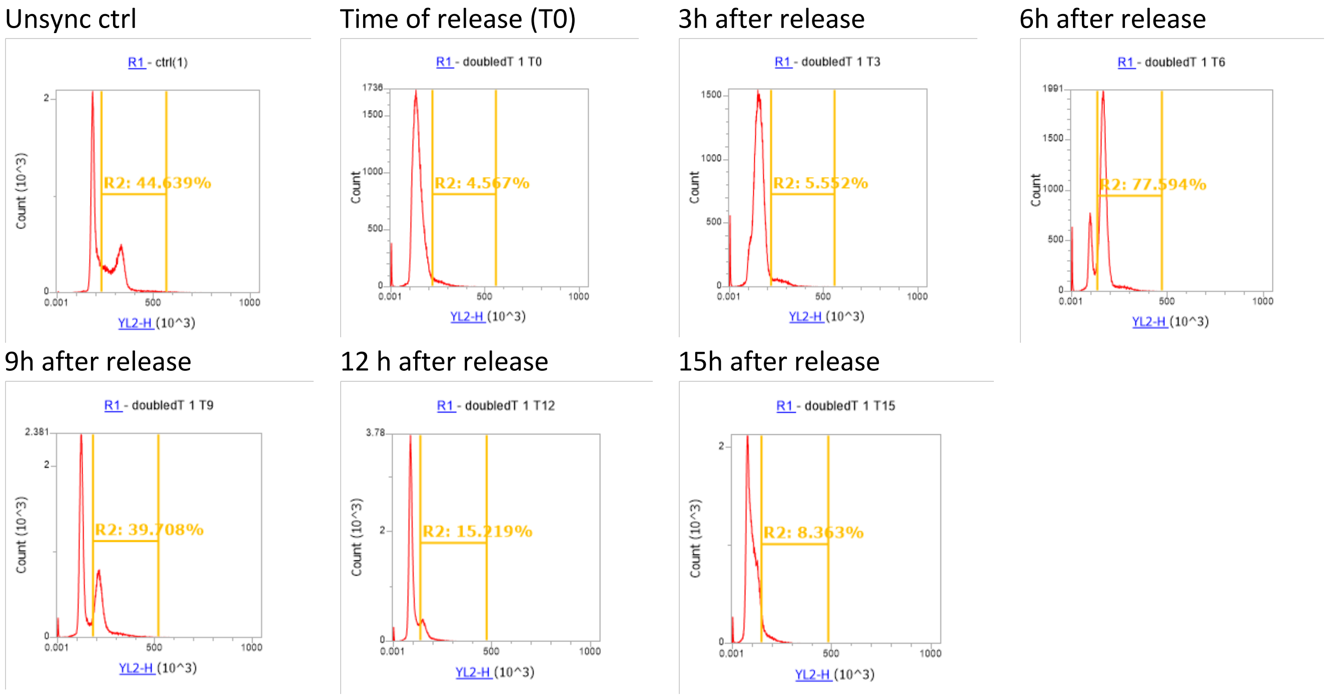

Fig. S9. FACS time-course measuring DNA content in HEK293T after release of second thymidine block. Same as Fig. S8, but after release of the second thymidine block.

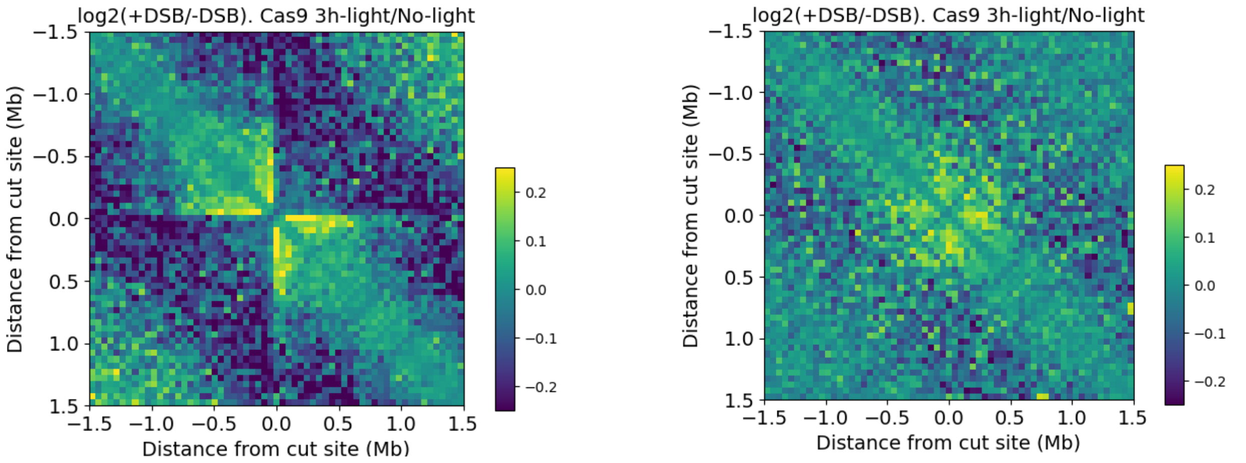

Fig. S10. Cas9-induced changes in Hi-C contacts in HEK293T cells synchronized in S/G2 (left) and G1 (right) phases of the cell cycle. Biological replicate of Fig. 2f, g, main text. Data was obtained, analyzed and represented as in Fig. 2f, g.

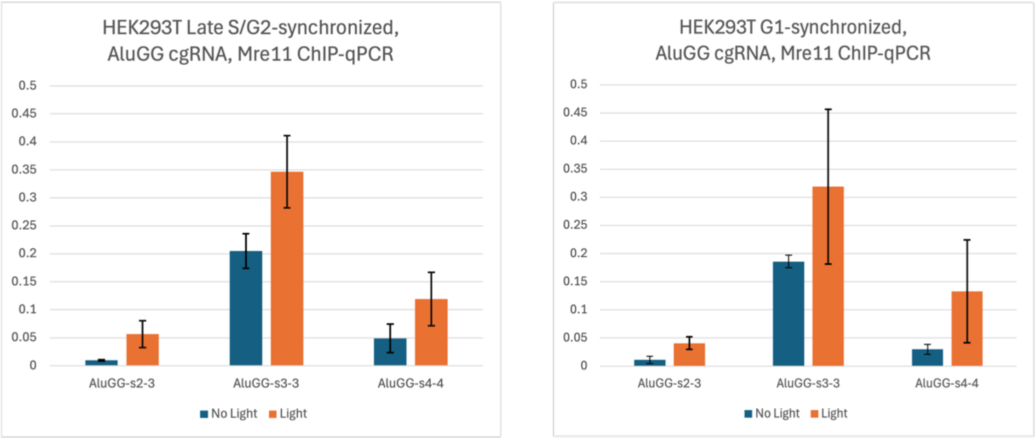

Fig. S11. MRE11 ChIP-qPCR in late S/G2 (left) and G1 (right) synchronized HEK293T cells subjected to AluGG cgRNA DSBs. ChIP enrichment was quantified as percentage of input chromatin in cell subjected to light and no-light. Three specific AluGG cut sites were assayed (see methods section for qPCR details). ChIPs and qPCRs were done in biological replicates. Errors are the standard deviation. G1-synchronized cells showed similar MRE11 enrichment upon DSB induction compared to late S/G2 cells.

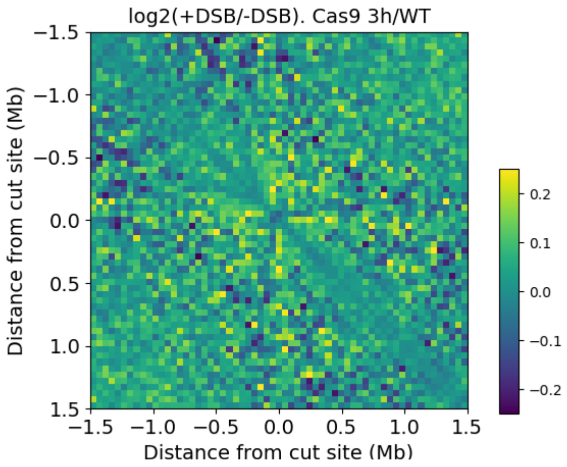

Fig. S12. Cas9-induced changes in Hi-C contacts in cells exposed to mirin, second replicate. Biological replicate of Fig. 1h, main text.

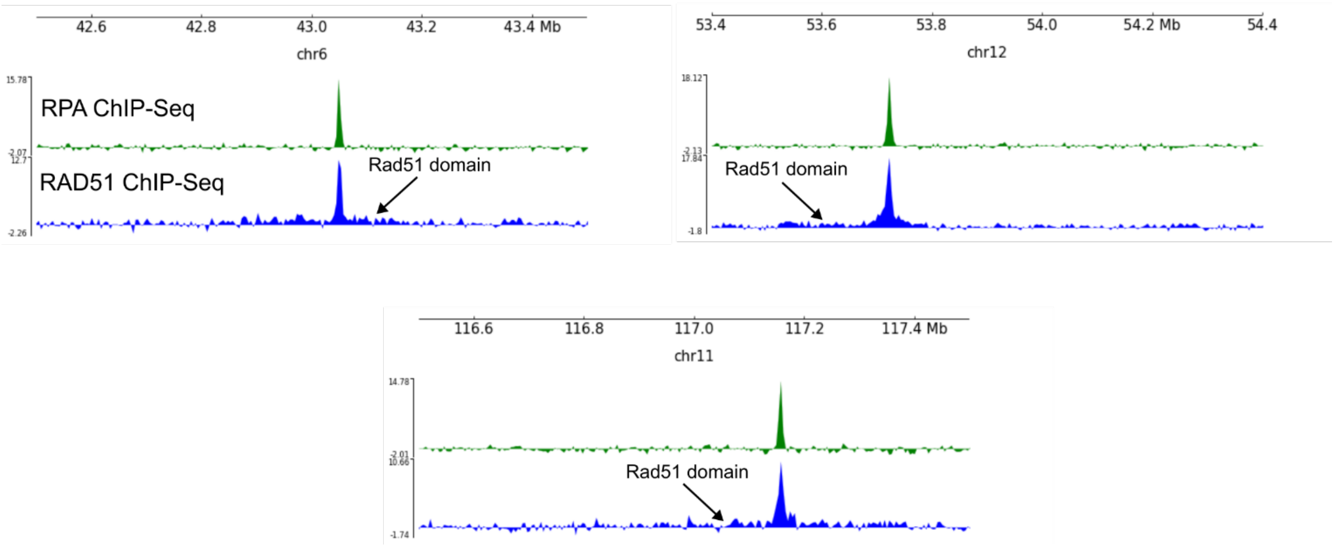

Fig. S13. Snapshots of RPA and RAD51 ChIP-Seq enrichment at three select AluGG sites in HEK293T cells treated with AluGG-Cas9 RNP.

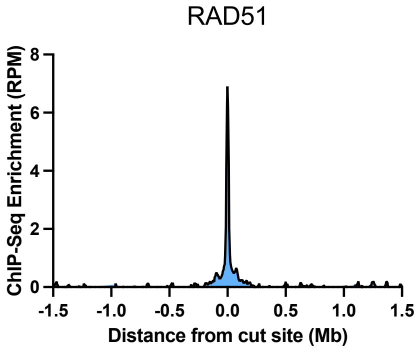

Fig. S14. Average, Cas9-induced RAD51 enrichment using antibody #2. Data analysis and plot details are the same as in Fig. 3d, main text.

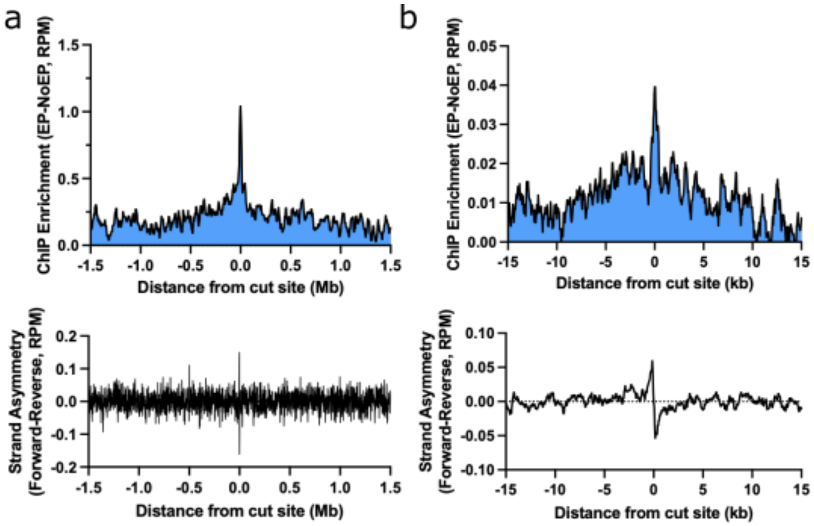

Fig. S15. Strand-specific ChIP-Seq, 2nd replicate. Data analysis and plotting details are the same as in Fig. 3 f, g, main text.

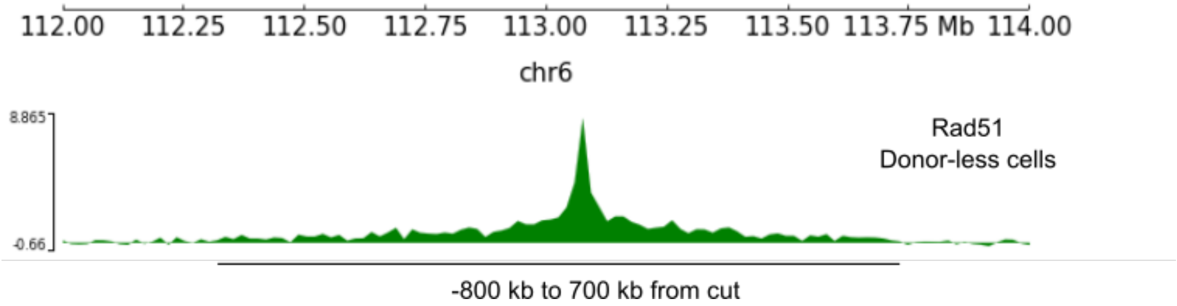

Fig. S16. Rad51 ChIP-Seq profile at *Rosa26* in mES donor-less cells. Data analysis and plotting details as in Fig. 4c.

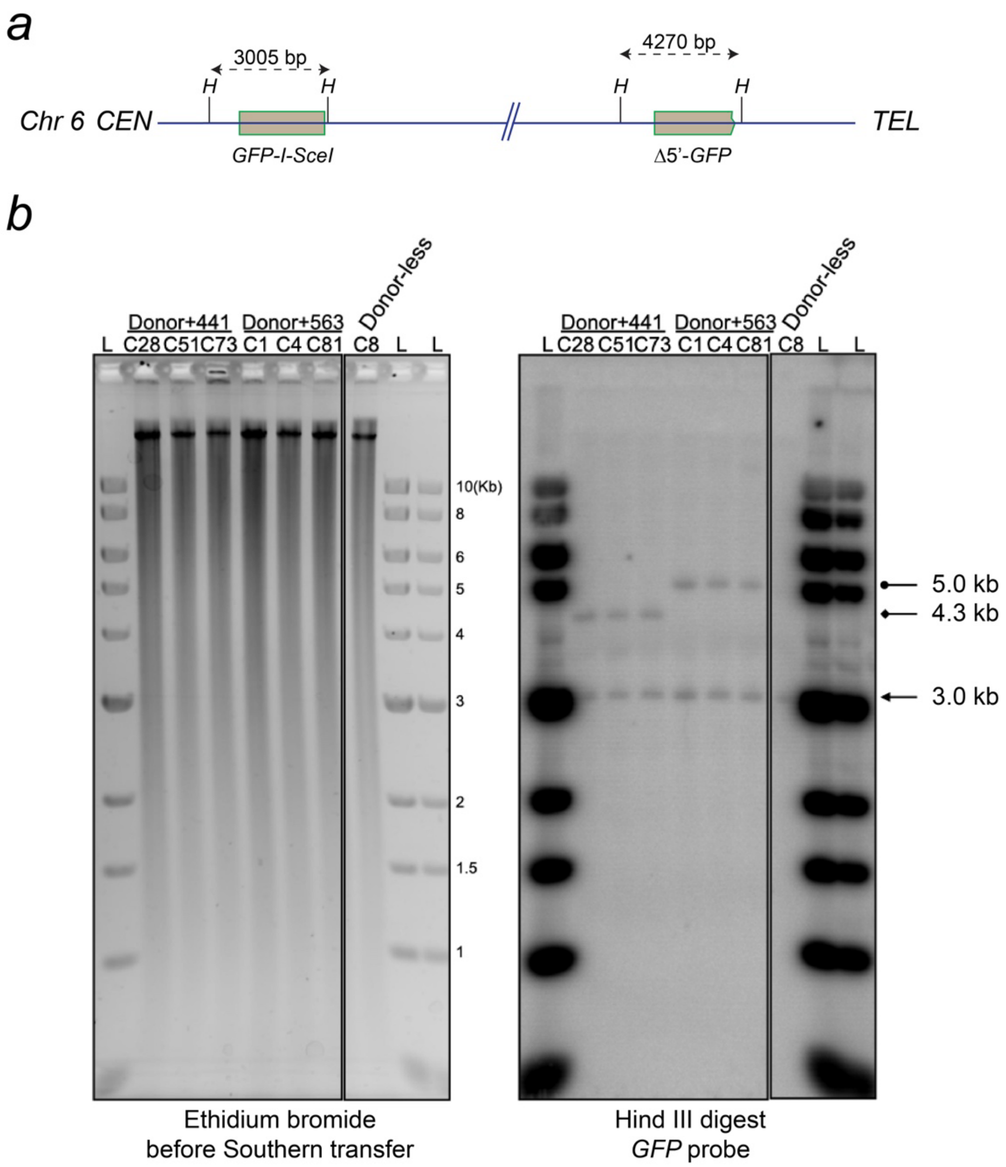

Fig. S17. **Identification of single-copy integrants of Donor+441 and Donor+563 clones.** Conventional Southern blotting (*GFP* probe) following HindIII digestion of gDNA for three correctly-targeted clones of Donor+441 and of Donor+563. ***a***. Cartoon depicting *GFP*-hybridizing HindIII (H) restriction fragments for Donor+441 clones. Only *GFP* elements are shown. *GFP-I-SceI* is detected as a 3005 bp fragment, while the +441 *Δ5'-GFP* donor is detected as a fragment of 4270 bp. The equivalent *Δ5'-GFP* fragment for Donor+563 is 4978 bp (not shown). ***b***. Southern blot analysis of Donor+441 clones #28, #51 and #73 and Donor+563 clones #1, #4, and #81. L: MW ladder. Invariant 3.0 kb band of *GFP-I-SceI* is seen in all clones, including in the 'donorless' clone lane (labeled 'C8'). Note that the targeting bands of 4.3 kb (Donor+441 clones) and 5.0 kb (Donor+563 clones) are of the same intensity as the invariant 3.0 kb band. This shows that these clones contain a single copy of the *Δ5'-GFP* donor at the planned site of integration. However, the question of whether the *Δ5'-GFP* donor is on the same chromosome as the *GFP-I-SceI* heteroallele ('targeting *in cis*') or on the homolog ('targeting *in trans*') cannot be resolved by conventional Southern blotting.

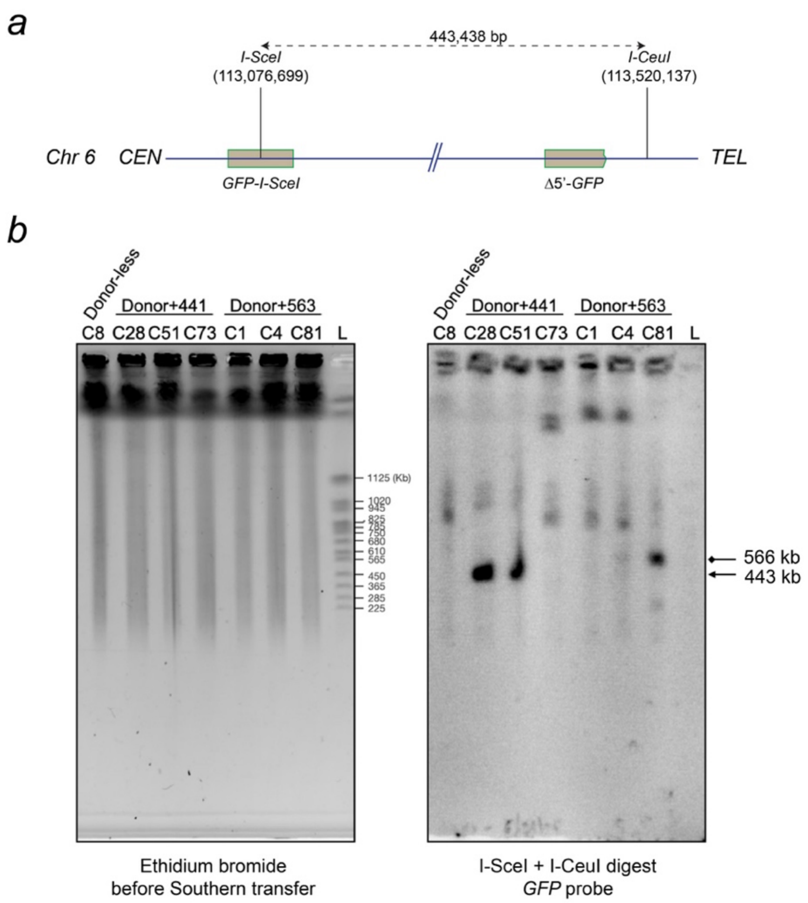

Fig. S18. **Differentiation between *Δ5'-GFP* donor targeting *in cis* vs. *in trans*.** The targeted *GFP* donor could be located on either the allele of Chr 6 that contains the *GFP-I-SceI* heteroallele at *Rosa26* (targeting *in cis*), or the homolog that is unmodified at *Rosa26* (targeting *in trans*). We used pulsed field gel electrophoresis (PFGE) to distinguish between these two outcomes for the single-copy targeted clones shown in **Fig. S17**. This method made use of a restriction site for the rare-cutting restriction endonuclease, I-CeuI, which we placed at the inner edge of the telomeric targeting arm of the Donor *Δ5'-GFP* targeting construct. In this way, following successful targeting, digestion with I-SceI + I-CeuI will generate a *GFP*-hybridizing fragment of ~443 kb if the clone is targeted *in cis*, but no fragment of this size if the donor is targeted *in trans* (i.e., to the homolog) ***a***. Cartoon depicting *GFP*-hybridizing fragments for Donor+441 clones, following restriction digestion of gDNA with I-SceI + I-CeuI. Only *GFP* elements are shown. Positions of *I-SceI* and *I-CeuI* sites on Chr 6 map (containing HR reporter elements) are in parentheses. *cis*-targeted Donor+441 clones generate a fragment of predicted size 443,438 bp. The equivalent fragment size for a *cis*-targeted Donor+563 clone is 565,713 bp (not shown). ***b***. PFGE-Southern blot analysis of Donor+441 clones #28, #51 and #73 and Donor+563 clones #1, #4, and #81. L: *S. cerevisiae* chromosomal MW ladder. Note *GFP*-hybridizing fragments of correct size for Donor+441 clones #28 and #51, but not #73; and for Donor+563 clone #81, but not #1 or #4. Therefore, Donor+441 clones #28 and #51 and Donor+563 clone #81 are targeted *in cis*, while Donor+441 clone #73 and Donor+563 clones #1 and #4 are targeted *in trans*.

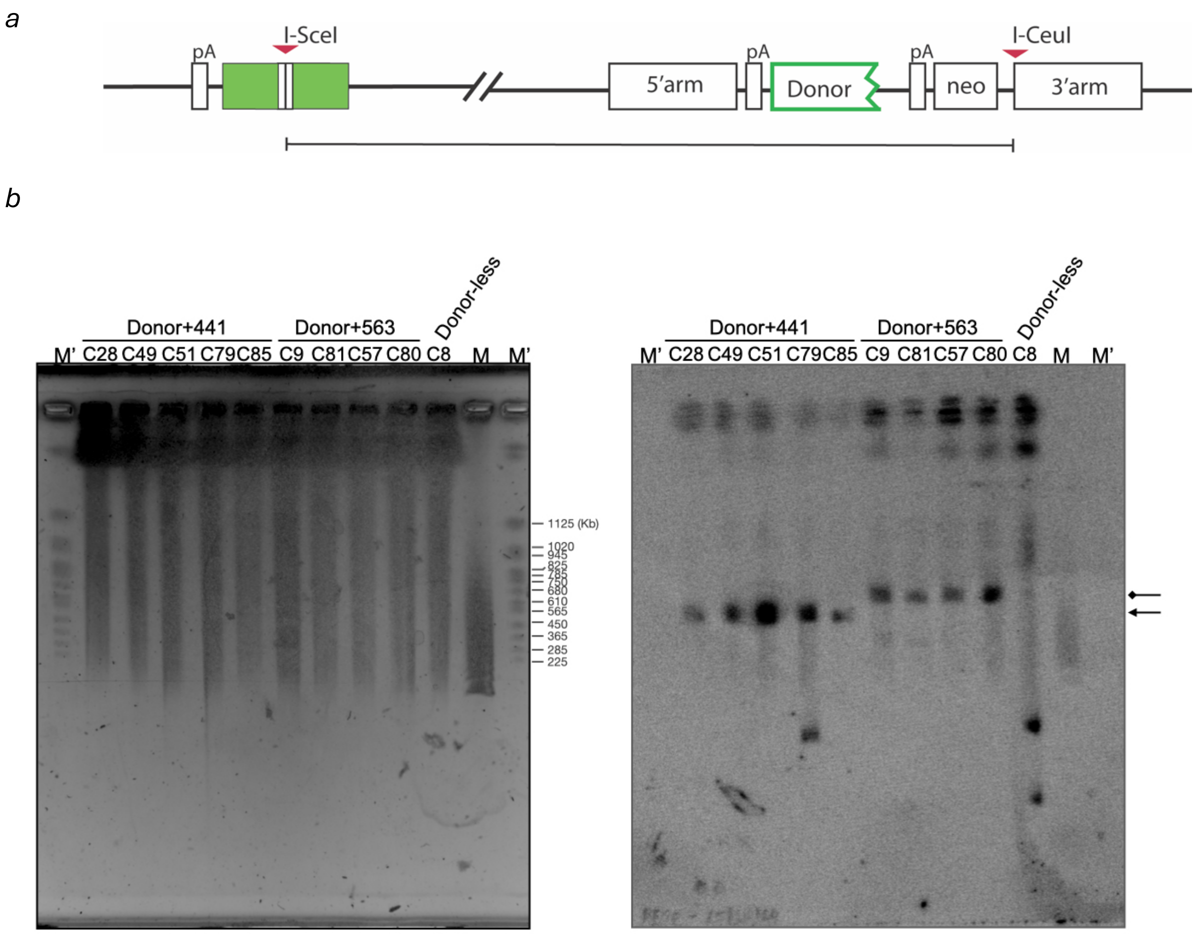

Fig. S19. PFGE analysis of additional *Δ5'-GFP* Donor+441 and Donor+563 targeted clones. ***a.*** Schematic depicting HR reporter. Promoters and other reporter elements not shown. **neo**: neomycin resistance gene. **pA**: polyadenylation signal. At *Rosa26* locus, full length *GFP* heteroallele is disrupted by a single *I-SceI* site. The *Δ5'-GFP* donor was targeted either +441 or +563 kb telomeric to *GFP-I-SceI* using CRISPR-Cas9-assisted homologous recombination. The ~500 bp targeting arms of the *Δ5'-GFP* donor targeting vector are denoted 5' arm (centromeric) and 3' arm (telomeric) respectively. The 3' arm contains a single *I-CeuI* site at its inner edge, as shown. Combined I-SceI and I-CeuI digestion generates *GFP*-hybridizing fragments of ~443 kb (Donor+441) or ~566 kb (Donor+563). See also **Fig. S18**. ***b.*** Pulsed-field gel electrophoresis ethidium bromide-stained agarose gel prior to Southern transfer (left) and Southern blot analysis with *GFP* probe (right) of multiple independent *cis*-targeted Donor+441 and Donor+563 clones. gDNA in agarose plugs from each clone was digested with I-SceI and I-CeuI (see Methods). M: *S. cerevisiae* chromosomal DNA marker. M': Lambda PFG ladder. Arrowheads mark the location of the expected *GFP*-hybridizing ~443 kb and ~566 kb fragments of *cis*-targeted Donor+441 and Donor+563 clones, respectively. Clone numbers are indicated. Donor-less clone #8 serves as negative control. Donor+441 clone #79 was excluded because of additional off-size *GFP*-hybridizing fragment. *Note*: Donor+441 clones #28, #49, #51 and #85 and Donor+563 clone #81 were found by conventional Southern blotting to harbor a single targeted copy of the *Δ5'-GFP* donor (**Fig. S17** and data not shown). *Note*: Donor+563 clones #9, #57 and #80 revealed 2x intensity of *Δ5'-GFP* donor-hybridizing fragments by conventional Southern blotting (not shown), suggesting that the *Δ5'-GFP* donor had been targeted to both alleles of the target locus. These clones were therefore excluded from further analysis.

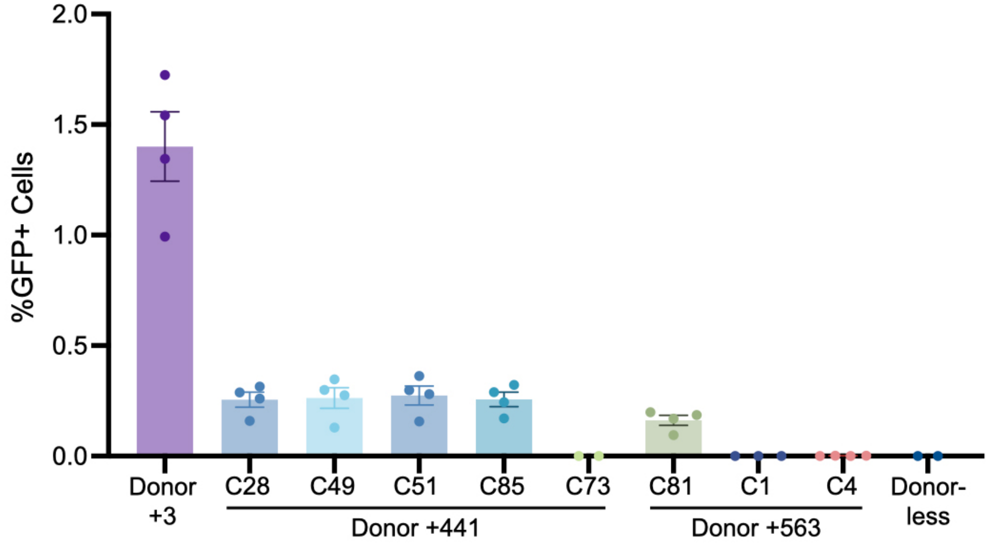

Fig. S20. Donor targeting *in cis* or *in trans* predicts efficiency of I-SceI-induced HR. I-SceI-induced HR (measured as induction of GFP^{+} HR products) in a panel of single copy-targeted Donor+441 and Donor+563 clones. HR was quantified as described in Materials and Methods. Donor+3 and Donorless clones serve as positive and negative controls, respectively. Note readily detectable HR in Donor+441 clones #28, 49, 51 and 85 (independent single-copy *cis*-targeted clones) but not in #73 (single-copy *trans*-targeted clone). Similarly, HR is readily detectable in Donor+563 clone #81 (single-copy *cis*-targeted clone) but not in clones #1 or #4 (independent single-copy *trans*-targeted clones). Donor targeting *in trans* generates levels of HR that are below the level of detection in the assay shown. Parts of this dataset are also shown in **Fig. 3k**.

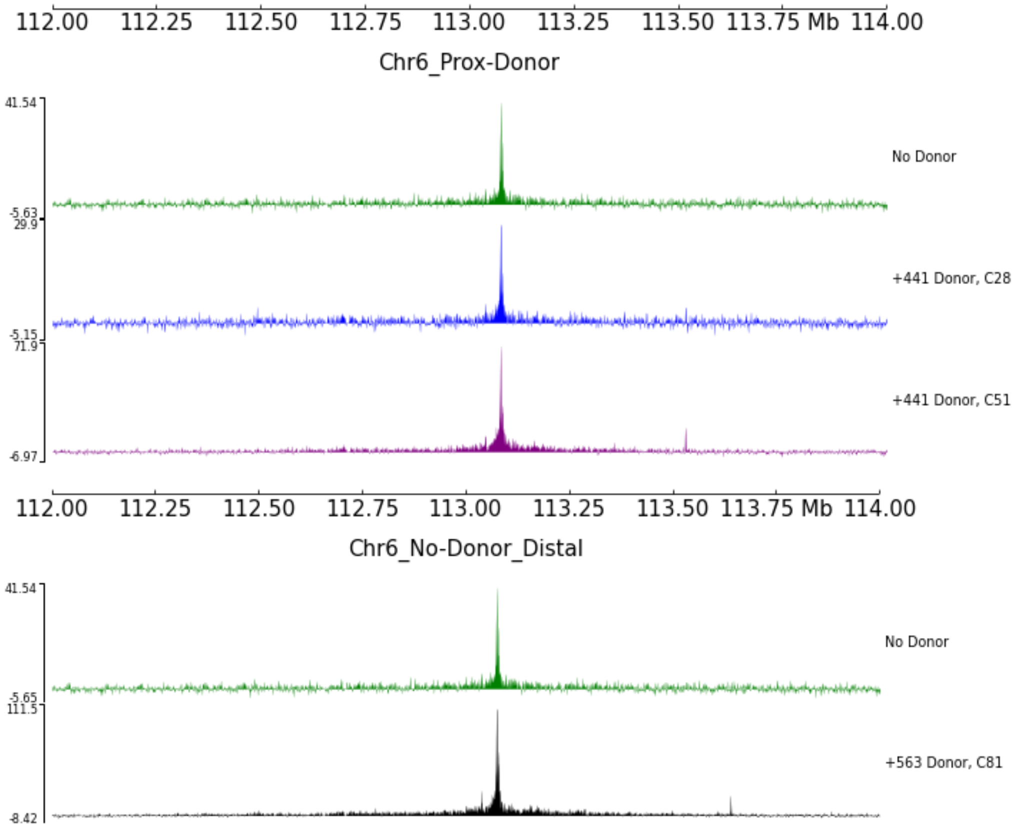

Fig. S21. Rad51 ChIP-Seq in donor-less and distal-donor cells. Second replicates. Top tracks, ChIP-Seq on cells with no-donor (green) and two +441 clones (blue and purple) aligned with a mm10 genome containing a donor at +441 kb from DSB. Bottom tracks, ChIP-Seq on cells with no-donor (green) and a +563 clone (black) aligned to a genome with the donor at +563 kb. Donor-less ChIP-Seq profiles showed no enrichment at the expected donor location.

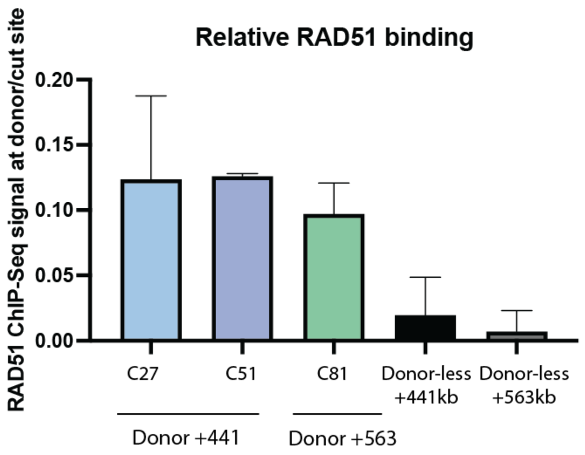

Fig. S22. Relative RAD51 at donor site compared to cut site. RAD51 enrichment was computed in a window of 6kb centered at the donor site and a window of 6 kb centered at the cut site. The ratio between the two was then calculated. From left to right: relative RAD51 signal donor/DSB in two +441kb clones, one +563kb clone and the donor-less clone evaluated at +441 kb and +563 kb. Shown are the average over two biological replicates, error bars represent the standard deviation.

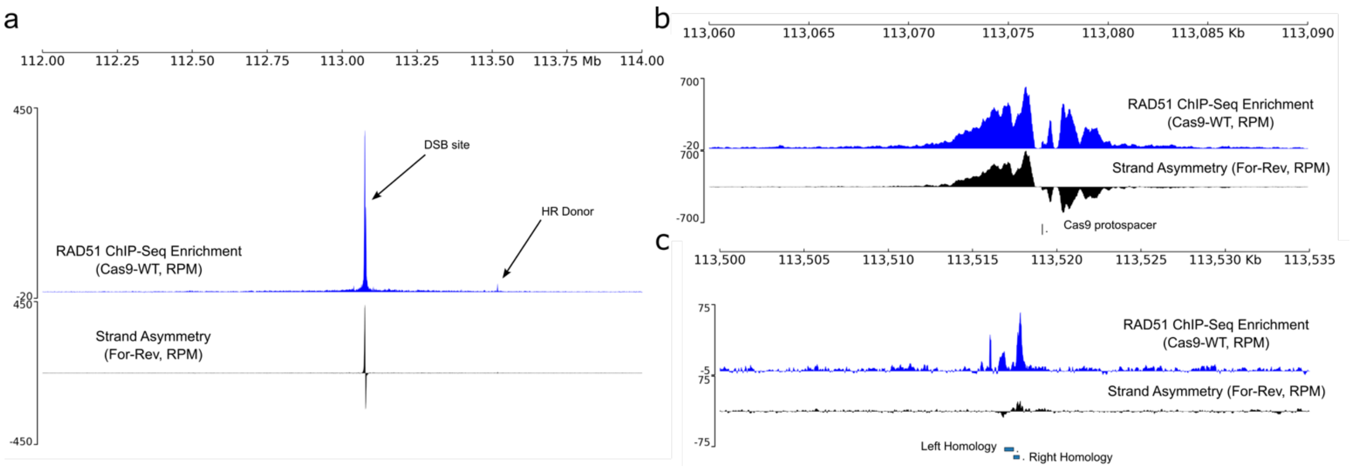

Fig. S23. Strand-specific RAD51 ChIP-Seq on mES clones with +441 kb donor. a, Top panel: RAD51 ChIP-Seq enrichment profile was obtained as the difference in normalized RAD51 ChIP-Seq reads in the Cas9-treated and the untreated cells. Bottom panel: strand asymmetry of the RAD51 ChIP-Seq signal in Cas9 treated cells was obtained by subtracting the forward and the reverse reads. Bin size: 1 kb. b, Zoom into DSB region showing RAD51 ChIP-Seq signal (Cas9-WT), strand asymmetry profile (Cas9 treated only) and the location of the Cas9 protospacer marking the DSB site. c, Zoom of the donor region. Same as b, but zoomed at the donor region and showing the left and right region of homology at the HR donor. Bin size: 15 bp (b, c).

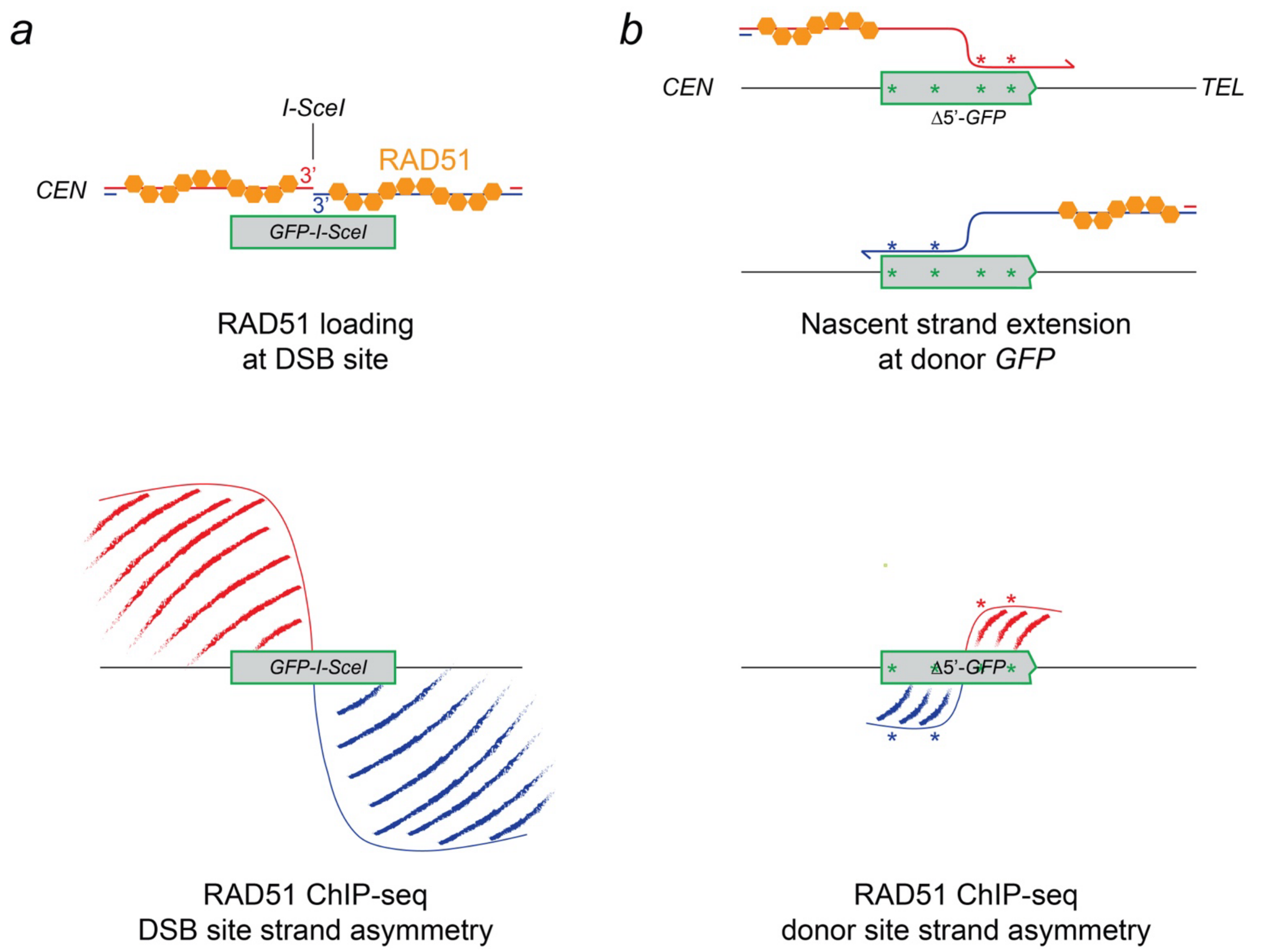

Fig. S24. RAD51 ChIP-seq strand asymmetries over the DSB site and over the HR donor site. a. Strongly asymmetric patterns of RAD51 ChIP-Seq at DSB site reflect RAD51 loading onto long 3' ssDNA tails following DNA end resection. **CEN:** centromeric. **TEL:** telomeric. **Red:** (+) strands. **Blue:** (–) strands. **Orange hexagons:** RAD51. Not to scale. **b.** Hypothetical explanation of the observed RAD51 ChIP-Seq strand asymmetry at the Δ5'-*GFP* donor site. Not to scale. Note that the polarity of RAD51 strand asymmetry over the *Δ5'-GFP* heteroallele is the opposite to that seen over the *GFP-I-SceI* heteroallele. **Asterisks:** engineered silent mutations in *Δ5'-GFP* donor that enable accurate alignment calls during ChIP-Seq analysis of *GFP* sequences. **Half arrows:** nascent strand extension (DNA synthesis during gene conversion). D-loops not shown. **Orange hexagons:** RAD51. We speculate that RAD51 remains associated with ssDNA continuous with the nascent strand during gene conversion, enabling pull-down of the nascent strand during RAD51 ChIP-Seq. *Note*: the majority of RAD51 ChIP-Seq signals over the donor *Δ5'-GFP* heteroallele reveal no strand asymmetry. We interpret this dsDNA-associated RAD51 ChIP signal as evidence of prolonged association of the DSB site RAD51 filament with dsDNA at the Δ5'-*GFP* donor site during a successful homology search (not shown).

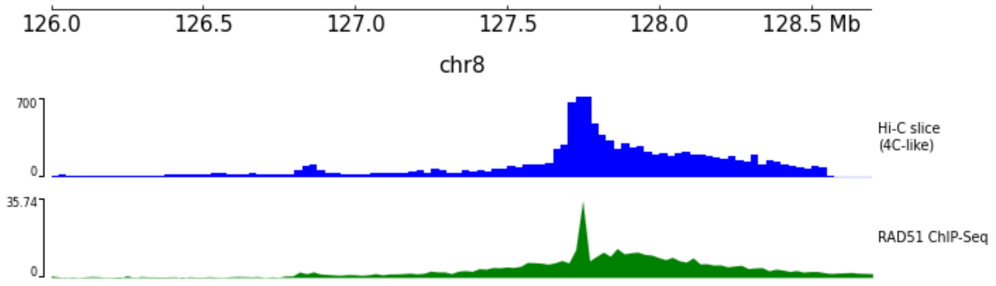

Fig. S25. RAD51 ChIP-Seq profile and 4C-like plot centered at MYC gene. Figure details as in Fig. 4c, main text.

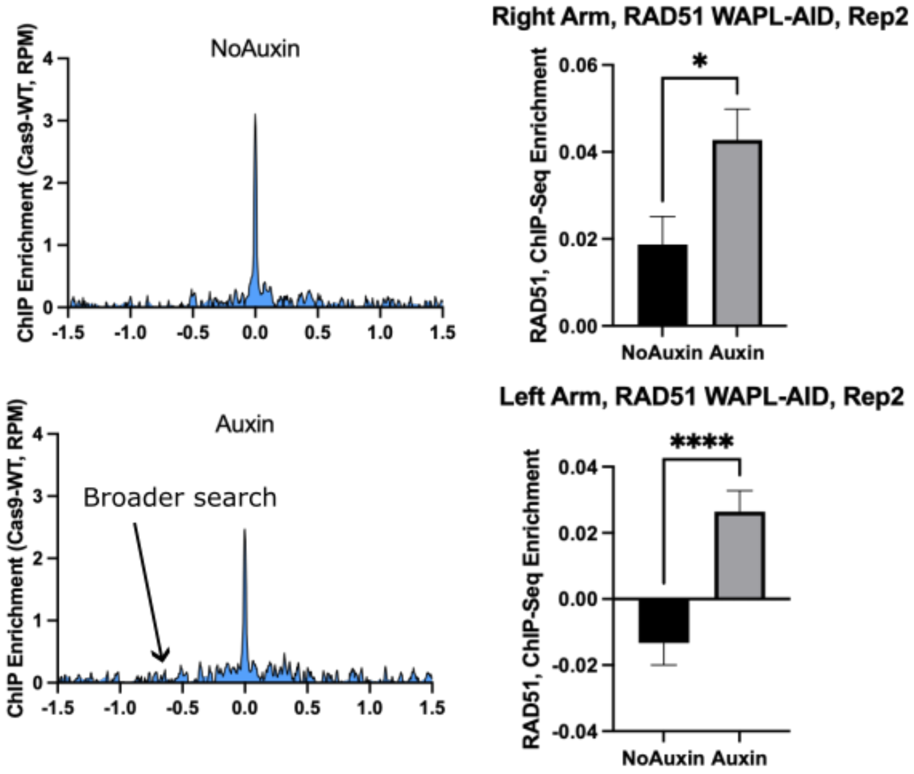

Fig. S26. RAD51 in WAPL-AID-HCT116 cells subjected to AluGG DSBs. Second biological replicate of Fig. 4 d-g.

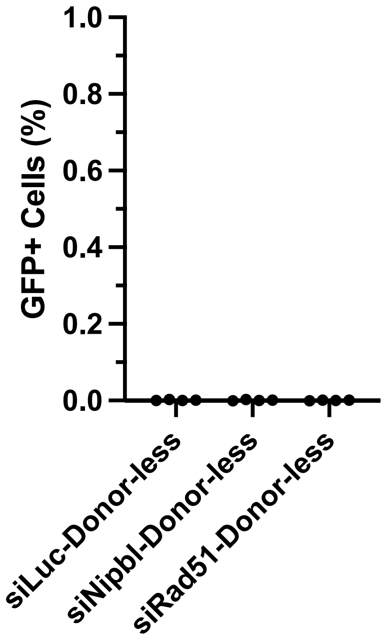

Fig. S27. HR-GFP assays in donor-less clone treated with luciferase, Nipbl or Rad51 siRNA.

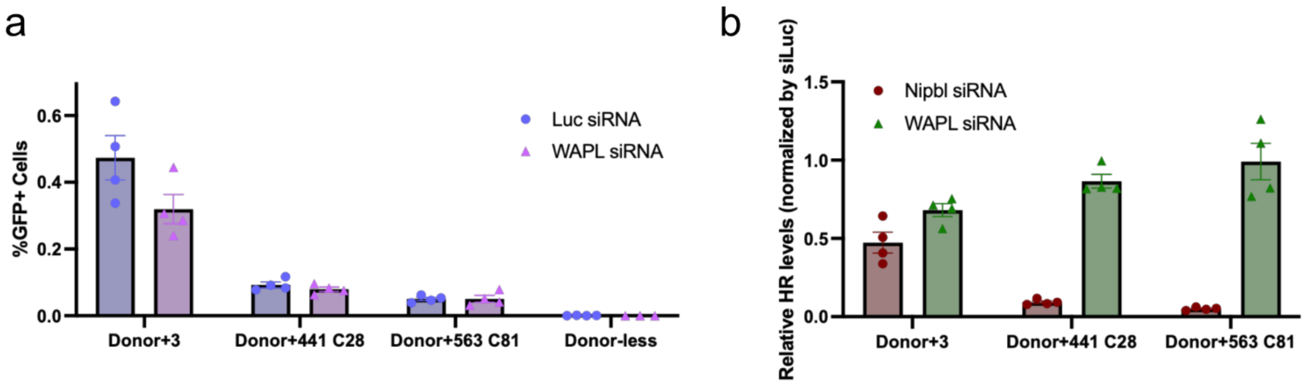

Fig. S28. Effect of WAPL degradation on HR. a, HR levels in HR-GFP clones with different DSB-donor distances (+3, +441, +563) and with no donor (donor-less) upon Luciferase or WAPL siRNA treatment. b, Relative HR levels (normalized by Luciferase siRNA) in Donor+3, Donor+441 and Donor+563 clones treated with Nipbl (red circles) or WAPL (green triangles) siRNA. Shown is the average of four biological replicates. Error bars are the standard error of the mean.

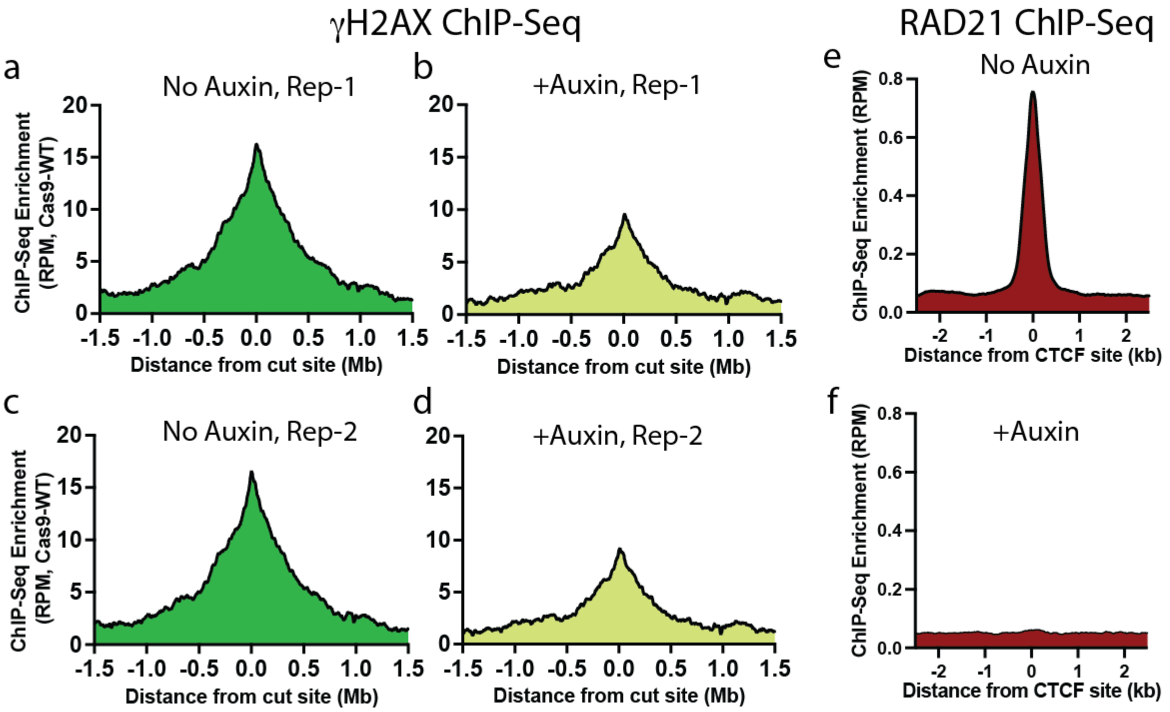

Fig. S29. γH2AX ChIP-Seq (a-d) and RAD21 ChIP-Seq (e, f) in RAD21-AID cells. γH2AX ChIP-Seq enrichment (Cas9-WT) in HCT116-RAD21-AID2 cells with no drug (a, c) or treated with auxin (1 uM of 5-Ph-IAA) 1h before RNP nucleofection (c, d). Enrichment profiles were obtained as in Fig. 1e, main text. RAD21 ChIP-Seq in HCT116-RAD21-AID2 cells with no drug (e) or 4h after auxin treatment (f) averaged around 1000 CTCF sites.

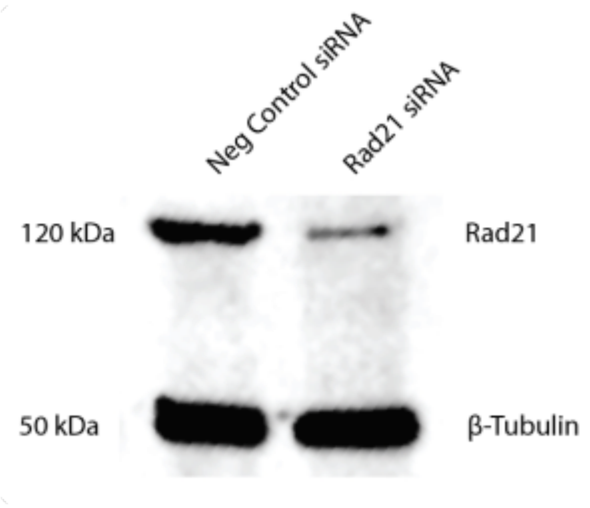

Fig. S30. Western blot on HEK293T treated with Neg Control and Rad21 siRNA. RAD21 depletion levels were estimated to be ~70%.

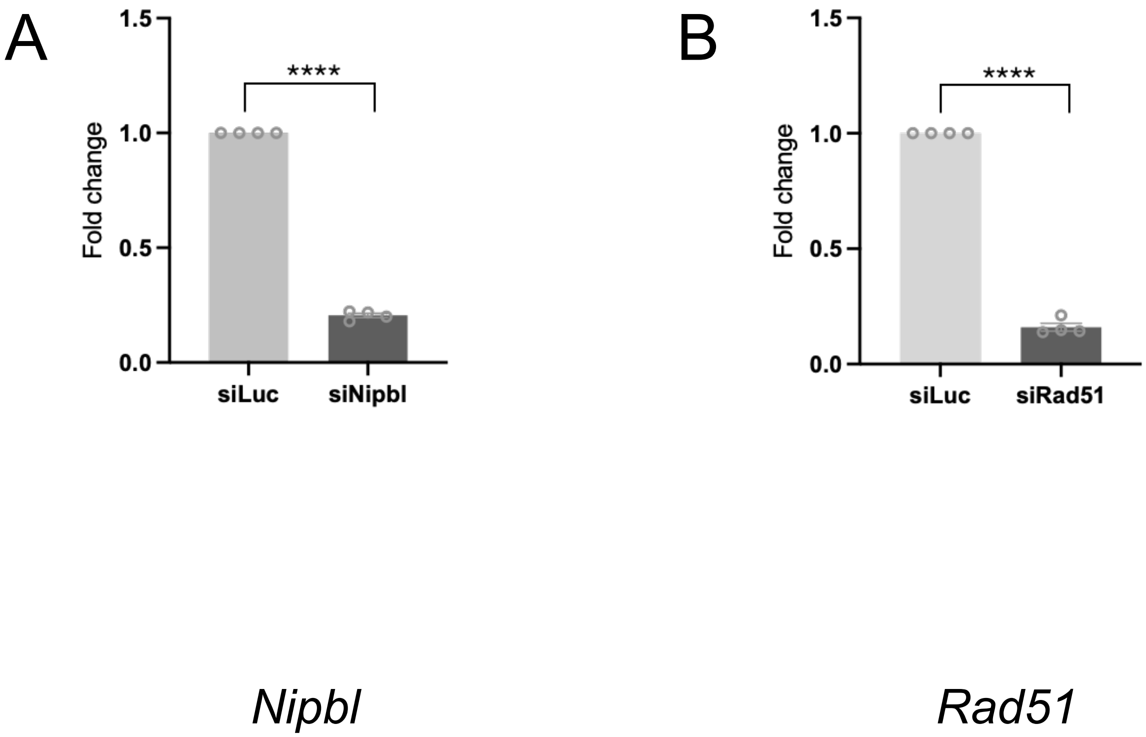

Fig. S31. **Depletion of target mRNAs by si*Nipbl* and si*Rad51*.** RT–qPCR analysis of abundance of *Nipbl* mRNA (panel **A**) and *Rad51* mRNA (panel **B**) in Clone# 8, following siRNA transfections shown. Data normalized to *Gapdh* mRNA abundance and expressed as fold-change relative to non-targeting si*Luc* sample (specific for firefly Luciferase mRNA) and analyzed using the $2^{-\Delta\Delta Ct}$ method. n=4 technical replicates. Analysis by Student's t-test. ****: P= <0.0001.

Supplementary Tables.

Table S1: crRNA and tracrRNA sequences.

Name	crRNA Sequences (5' to 3')<br>Red marks the RNA sequences hybridizing with target DNA
AluGG	CCU GUA GUC CCA GCU ACU GGG UUU UAG AGC UAU GCU
Caged AluGG	CC[NPOM-dT] G[NPOM-dT]A G[NPOM-dT]C CCA GCU ACU GGG UUU UAG AGC UAU GCU GUU UUG
ACTB	GCU AUU CUC GCA GCU CAC CAG UUU UAG AGC UAU GCU GUU UUG
MYC	GUA AUU CCA GCG AGA GGC AGG UUU UAG AGC UAU GCU GUU UUG
Rosa26	GGA UAA CAG GGU AAU CAA GGG UUU UAG AGC UAU GCU GUU UUG
**Name**	**tracrRNA sequence (5' to 3')**
tracrRNA	AGCAUAGCAAGUUAAAAUAAGGCUAGUCCGUUAUCAACUUGAA AAAGUGGCACCGAGUCGGUGCUUU

Table S2: antibodies used in this study.

Target	Company	ID	Quantity used per ChIP (uL)
Rad51, aB1	Novus	NB100-148	7.5
Rad51, aB2	Millipore Sigma	PC130	7.5
Mre11	Novus	NB100-142	3
NIPBL	Proteintech	18792-1-AP	9
RPA2	Thermo Fisher Scientific	MA1-26418	50
53BP1	Novus	NB100-305	3
yH2AX	Abcam	ab81299	3
Rad21	Abcam	ab992	4
pSMC1	Abcam	ab81306	10

Supplementary References

1. Y. Liu *et al.*, Very fast CRISPR on demand. *Science* **368**, 1265-1269 (2020).

2. N. A. Willis, A. Panday, E. E. Duffey, R. Scully, Rad51 recruitment and exclusion of non-homologous end joining during homologous recombination at a Tus/Ter mammalian replication fork barrier. *PLOS Genetics* **14**, e1007486 (2018).

3. Suhas S. P. Rao *et al.*, A 3D Map of the Human Genome at Kilobase Resolution Reveals Principles of Chromatin Looping. *Cell* **159**, 1665-1680 (2014).

4. N. A. Willis *et al.*, BRCA1 controls homologous recombination at Tus/Ter-stalled mammalian replication forks. *Nature* **510**, 556-559 (2014).
